# Supervised DNA Barcodes species classification: analysis, comparisons and results

**DOI:** 10.1186/1756-0381-7-4

**Published:** 2014-04-11

**Authors:** Emanuel Weitschek, Giulia Fiscon, Giovanni Felici

**Affiliations:** 1 Department of Engineering, Roma Tre University, Via della Vasca Navale, 79, 00146 Rome, Italy; 2 Institute of Systems Analysis and Computer Science Antonio Ruberti, National Research Council, Viale Manzoni, 30, 00185 Rome, Italy; 3 Department of Computer, Control, and Management Engineering, Sapienza University, Via Ariosto, 25, 00185 Rome, Italy

**Keywords:** DNA Barcoding, Supervised classification methods, Species identification

## Abstract

**Background:**

Specific fragments, coming from short portions of DNA (e.g., mitochondrial, nuclear, and plastid sequences), have been defined as DNA Barcode and can be used as markers for organisms of the main life kingdoms. Species classification with DNA Barcode sequences has been proven effective on different organisms. Indeed, specific gene regions have been identified as Barcode: *COI* in animals, *rbcL* and *matK* in plants, and *ITS* in fungi. The classification problem assigns an unknown specimen to a known species by analyzing its Barcode. This task has to be supported with reliable methods and algorithms.

**Methods:**

In this work the efficacy of supervised machine learning methods to classify species with DNA Barcode sequences is shown. The Weka software suite, which includes a collection of supervised classification methods, is adopted to address the task of DNA Barcode analysis. Classifier families are tested on synthetic and empirical datasets belonging to the animal, fungus, and plant kingdoms. In particular, the function-based method Support Vector Machines (SVM), the rule-based RIPPER, the decision tree C4.5, and the Naïve Bayes method are considered. Additionally, the classification results are compared with respect to *ad-hoc* and well-established DNA Barcode classification methods.

**Results:**

A software that converts the DNA Barcode FASTA sequences to the Weka format is released, to adapt different input formats and to allow the execution of the classification procedure. The analysis of results on synthetic and real datasets shows that SVM and Naïve Bayes outperform on average the other considered classifiers, although they do not provide a human interpretable classification model. Rule-based methods have slightly inferior classification performances, but deliver the species specific positions and nucleotide assignments. On synthetic data the supervised machine learning methods obtain superior classification performances with respect to the traditional DNA Barcode classification methods. On empirical data their classification performances are at a comparable level to the other methods.

**Conclusions:**

The classification analysis shows that supervised machine learning methods are promising candidates for handling with success the DNA Barcoding species classification problem, obtaining excellent performances. To conclude, a powerful tool to perform species identification is now available to the DNA Barcoding community.

## Background

In 2003 Hebert et al. [1] proposed DNA Barcoding as a technique to identify species. Specific fragments, coming from short portions of mitochondrial, nuclear and plastid DNA, have been defined as *DNA Barcode* and can be used as markers for organisms of the main life kingdoms. The following gene regions are chosen as Barcodes: cytochrome C Oxidase subunit I (*COI*) for animals [2], *rbcL* and *matK* for plants [3], and the Internal Transcribed Spacer (*ITS*) for fungi [4].

Taxonomists identify biological specimens by morphological features, however in some tough cases the identification becomes complex even for experts. DNA Barcoding solves this problem, because it is able to distinguish species and identify specimens (also incomplete, damaged or immature ones) using a very short gene sequence, that can be easily obtained from tiny amounts of tissue.

It is now recognized that a DNA Barcode provides the sufficient information needed to classify a specimen to species, showing an high variability even among closely related species [5,6]. Thus, since 2004 the International Barcode Of Life project (*IBOL*) and the Consortium for the Barcode Of Life (*CBOL*) has promoted international initiatives devoted to the development of DNA Barcoding as a global standard for the identification of biological species, aiming to build up an online freely available sequence database (http://www.barcodinglife.org).

Species classification with DNA Barcode is used to assign an unknown specimen to a known species by analyzing its DNA Barcode sequence, and has been proven effective on different organisms [5,6]. It has been handled with several approaches. So far, the following taxonomy of *ad-hoc* methods has been used [7,8]:

(i) tree-based methods;

(ii) similarity-based methods;

(iii) character-based methods (also called “diagnostic methods”).

Tree-based methods assign unidentified Barcodes (*query*) to species based on their membership of clusters in a DNA Barcode tree. This approach can be achieved, for example, with Parsimony (i.e., PAR [9]), or Neighbor Joining (i.e., NJ [10]), or Bayesian Inference [11]. Similarity-based methods (e.g., BLAST [12], NN [13], and TaxonDNA [14]) assign *query* Barcodes to species based on how much DNA Barcode characters they have in common. Character-based methods (e.g., DNA-BAR [15], BLOG [7], CAOS [16], BRONX [17,18], PTIGS-IdIt [19], Linker [20], Alignment-free analytics [21]) rely on the presence/absence of particular characters in DNA Barcode sequences for identification, instead of using them all [8].

The DNA Barcode classification problem may be approached as a supervised machine learning problem in the following way [7]: given a *reference* library composed of DNA Barcode specimen sequences of known species and a collection of unknown DNA Barcode sequences (*query* set), recognize the latter into the species that are present in the library.

More formally, giventhe learning function is the following: *f*: *X* → *Y*, where *X* is the input space (the DNA Barcode sequences attributes, e.g., the sequence positions with their nucleotides assignments) and *Y* is the output space (the species labels in which input data has to be classified). In a supervised machine learning problem the user has to provide as input a *reference* library containing specimens with a priori known species membership. Based on this *reference* set, the machine learning software computes the classification model. Subsequently, the classification model can be applied to a *query* set which contains specimens that require classification. The *query* set can contain *query* specimens with unknown species membership or, alternatively, specimens that also have a priori known species membership, allowing verification of the specimen classifications correctness [7]. To obtain reliable results the *reference* set has to be composed of a sufficient number of specimen sequences for each species (our experiments show that at least 4 specimens per species are necessary to obtain a reliable classification rate), and the sequences of each species have to include possibly all the nucleotide polymorphisms (variations). Consequently, the *query* set has to comprise only specimens from the same species that are present in the *reference* library. In general, *reference* and *query* sets are provided separately; if only one dataset is provided, it can be randomly divided over *reference* and *query* data in order to test the efficacy of the method. The ratio of the number of specimens in the *reference* and *query* dataset depends on the number of specimens and usually a reasonable choice is a 80–20 percentage split.

(i) a set of training examples (in the following referred as a *reference* set) containing specimens with a priori known species membership and

(ii) a test set (in the following referred as a *query* set) containing specimens which require classification,

The paper [13] includes a high level description of some supervised machine learning methods (Nearest Neighbor, CART, Random Forest and Kernel Functions), but an analysis framework and software are not provided.

In this work the efficacy of supervised machine learning methods to classify species with DNA Barcode sequences is shown, through the performance comparison with respect to *ad-hoc* DNA Barcode analysis methods. The Weka machine learning software [22], which includes a collection of supervised classification methods, is adopted to address the task of DNA Barcode analysis. Different types of classifiers (trees, rules, lazy learners, Bayesian and functions) are tested on public available synthetic and empirical datasets belonging to the animal, plant, and fungus kingdoms. In particular, the function-based method Support Vector Machines (SMO), the rule-based RIPPER (Jrip), the decision tree C4.5 (J48), and the Bayesian-based method Naïve Bayes are considered.

## Methods

### The supervised machine learning algorithms

The Weka tools collection for Machine Learning and Data Mining analysis [22] is used to approach the species classification problem with DNA Barcode sequences. Weka (Waikato Environment for Knowledge Analysis) is a Java open source package that collects the most popular algorithms to handle classification, numeric prediction, or clustering problems. Among the several packages collected in Weka, the “Weka.classifier” package includes the implementation of classification and prediction algorithms, comprising the most important “Classifier” class. The latter defines the structure of any schema of classification or prediction assessment and it is made up by two methods, *buildClassifier()* and *classifyIstances()*, whose implementation is necessary for all supervised machine learning algorithms.

In Table 1 all the available algorithms for classification, numeric prediction and clustering assessments are summarized. In greater detail, Table 2 highlights the Weka classifiers.

**Table 1:** Weka algorithms collection

**Classification**	**Prediction**	**Meta**	**Clustering**
Decision trees	Linear regression	Bagging	EM
Support Vector Machines	Model tree generators	Boosting	Coweb
Naïve Bayes	Instance-based learners	Stacking	-
Decision tables	Decision tables	Regression via classification	-
Locally weighted regression	Locally weighted regression	Classification via regression	-
Rule learners	Multi-layer perceptron	Cost sensitive classification	-

**Table 2:** Weka classifiers

**Kind of classification**	**Description**
**Bayes**	Bayesian network (e.g., Naïve Bayes)
**Functions**	Linear regression, neural networks, support vector machine
**Lazy**	Instance-based similarity (e.g., Nearest neighbor algorithm)
**Meta**	Bagging, boosting, stacking, regression through classification, classification through regression, cost sensitive classification
**Rules**	Rule-based classifiers
**Trees**	Tree classifier (e.g., decision tree)
**Mi**	Algorithms that handle multi-instance data
**Misc**	Various classifiers that do not fit in any another category

### Algorithms description

Among the Weka classifiers the following methods are tested on DNA Barcode sequences: (i) the function-based method Support Vector Machines (SMO) [23]; (ii) the rule-based RIPPER (Jrip) [24]; (iii) the decision tree C4.5 (J48) [25]; and (iv) the Bayesian-based method Naïve Bayes [26].

### SMO (SVM)

SMO [23] is the Weka implementation of the supervised learning function-based method Support Vector Machines (SVM). SMO is a discriminative classifier, that converts the *reference* data objects in multi-dimensional vectors and defines a separating hyperplane among the objects belonging to different classes, i.e., given labeled training data, the algorithm outputs an optimal hyperplane that separates the classes with the largest minimum distance. After a proper vector transformation, new objects from the *query* set are evaluated according to this separating hyperplane. For example, for a linearly separable set of 2D-points which belong to one of two classes, the SVM finds a separating line where points of the same class lie on the same half-space. One of the most relevant features of the SVM is to use a non-linear transformation of the input space in a very efficient way via a linear Kernel function. SMO performs usually with high classification accuracy, but its main drawback is that no human readable classification model is provided as output.

### Jrip (RIPPER)

Jrip (RIPPER) [24] imp lements a propositional rule learner, Repeated Incremental Pruning to Produce Error Reduction, which was proposed by William W. Cohen. The algorithm performs two main phases: the first one builds an initial set of rules and the second one optimizes the rule set *k* times (typically *k* is set to 2). Specifically, the classes are examined in increasing size and an initial set of rules for each class is generated using incremental reduced error pruning. Then, all the examples of a particular judgment in the training data are treated as a class, and a set of rules that covers all the members of that class is found. Thereafter, the algorithm proceeds to the next class, repeating the same procedure until all classes have been covered. This method is a good candidate for DNA Barcoding as it provides a classification model composed of logic rules for each species in the dataset, that can be used to compactly characterize the analyzed specimens.

### J48 (C4.5)

J48 [25] is a supervised classification method belonging to the decision trees family. In particular, it represents the Weka implementation of the decision tree algorithm C4.5, that greedily looks for the best split and the best feature at each node in terms of the information gain measure. A decision tree is a simple tree structure whose non-terminal vertices represent tests on one or more attributes, while the terminal ones reflect the results of the decision. The key advantages of decision trees are the following: (i) they are simple and easily convertible into a set of rules; (ii) both numerical and categorical data can be classified (even if the output attribute must be categorical); (iii) there are no a priori assumptions about the nature of the features (e.g., qualitative, quantitative, ordinal data). However, decision trees are unstable (i.e., variations in the training data can produce different set of attributes to be chosen) and generally multiple output attributes are not allowed. Also in this method a classification model is given as output (the decision tree), which can be easily read as a set of logic rules composed by sequence positions and nucleotide assignments.

### Naïve Bayes

Naïve Bayes [26] is a Bayesian-based classifier using estimator classes. It is one of the most practical learning methods often used when a large *reference* set is available.

A Bayesian Network (BN) is the joint probability distribution of a set of variables: based on the state of the observable variables and *a priori* probabilities represented by the edge in the relations between variables, the *a posteriori* probabilities of the unknown states are evaluated. In this way, BN can be considered as a tool of investigation and forecasting. Mathematically, the BN is a directed acyclic graph whose vertices are variables or states, while the edges are statistical dependencies between the variables and local probability distributions of the leaf vertices compared to the values of the parent ones. The absence of an edge between two vertices reflects their conditional independence. Contrarily, the presence of an edge from a vertex *X*_*i*_ to a vertex *X*_*j*_ can be explained as *X*_*i*_ is a direct cause of *X*_*j*_. The critical assumption of a Naïve Bayes classifier is the conditional independence of the set of attributes that describes each *x ∈ X* instance of the target function *f*: *X* → *Y*. Like in the SVM method, no clear classification model is provided to the investigator, who can only perform a “blind” assignment of specimen to species.

### Input, sequences conversion and output

**Figure 1:**
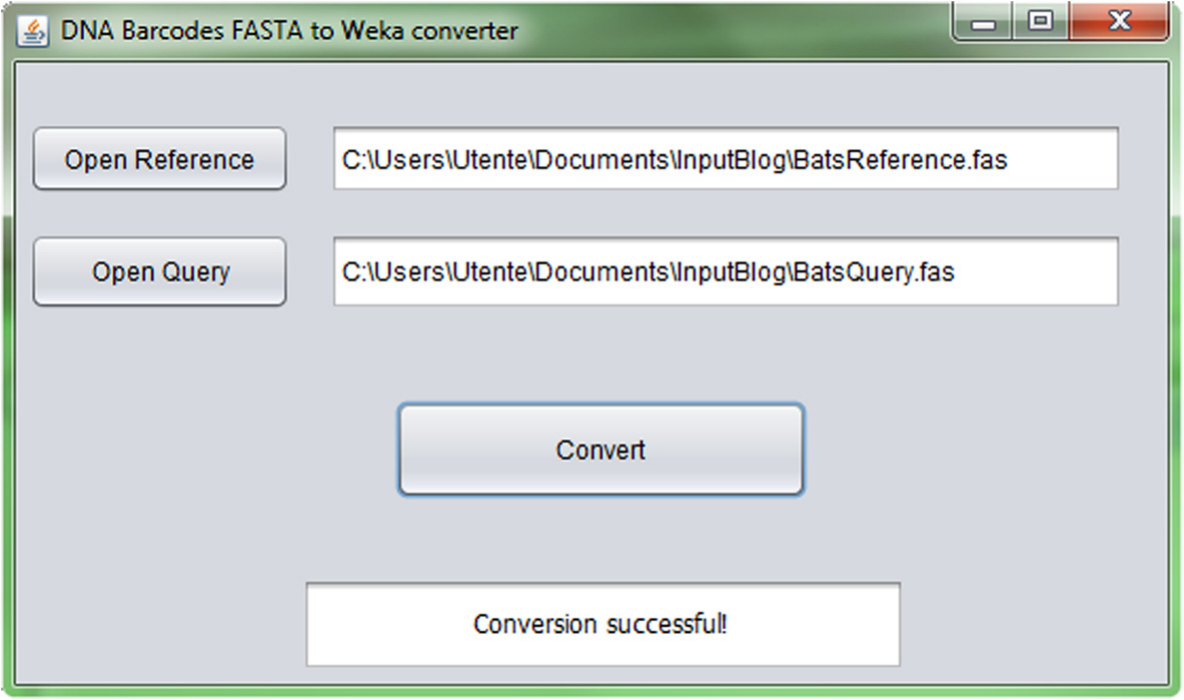
**DNA Barcode FASTA to Weka converter.** Screenshot of the converter tool developed to obtain the ARFF Weka format from the standard FASTA format of the DNA Barcode sequences.

DNA Barcode sequences are normally available and delivered in FASTA format, but Weka accepts as input its own file format called ARFF. Therefore, an integrated multi-platform (Windows, Linux and MacOS) Java program, available at http://dmb.iasi.cnr.it/supbarcodes.php, that converts the DNA Barcode FASTA sequences to the ARFF Weka format was developed and released (see Figure 1 for a screenshot).

Note that for supervised machine learning methods, the sequences have to be of the same region or pre-aligned to the same region before being processed (e.g., sub-segments of *COI* or *rbcL* coding genes) [7].

**Figure 2:**
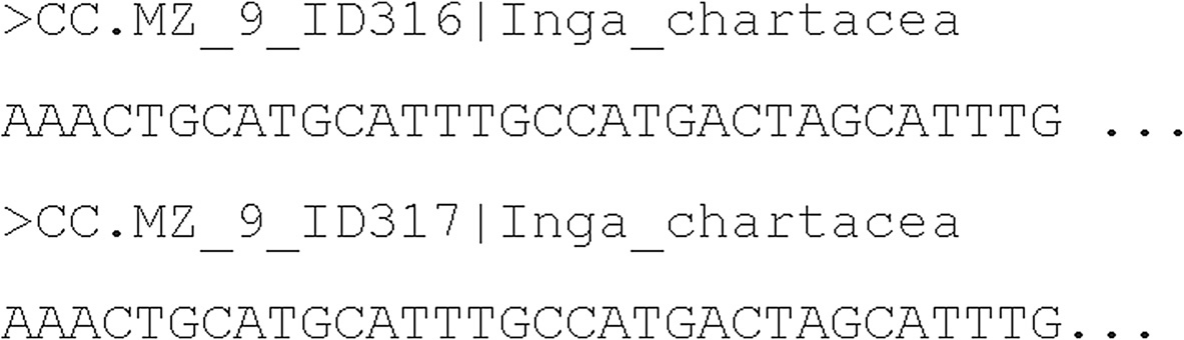
**Example of FASTA format.** The FASTA format is composed of two main parts: (i) the heading line of each sequence, starting with the character “>”, followed by the “specimen ID” and the “species name field” (dived by a vertical bar “|” ) and (ii) the nucleotide sequences (a string of A, C, G, T characters).

Input files are DNA Barcode sequence alignments in the standard FASTA format (*query* and *reference*), that need to be converted in the Weka input format (ARFF). The FASTA format is composed of a heading line for each sequence, that is formed by the starting character “>”, followed by the “specimen ID” and the “species name field” (divided by a vertical bar “|”). The following lines contain the nucleotide sequences (i.e., a string of A, C, G, or T characters). An example of FASTA format is given in Figure 2.

The software converts FASTA format into the ARFF Weka format. The latter is composed of two parts. The first part of the file includes the name of the dataset (starting with “@relation”), the heading line (starting with “@attribute”) for each attribute (i.e., sequence position), where the type of attribute is specified (e.g., numeric, a number, or categorical, a string of characters) and finally a complete list of the species enclosed in curly brackets. The second part (starting with “@data”) comprises a line for each specimen, that stores the attribute values separated by a comma.

**Figure 3:**
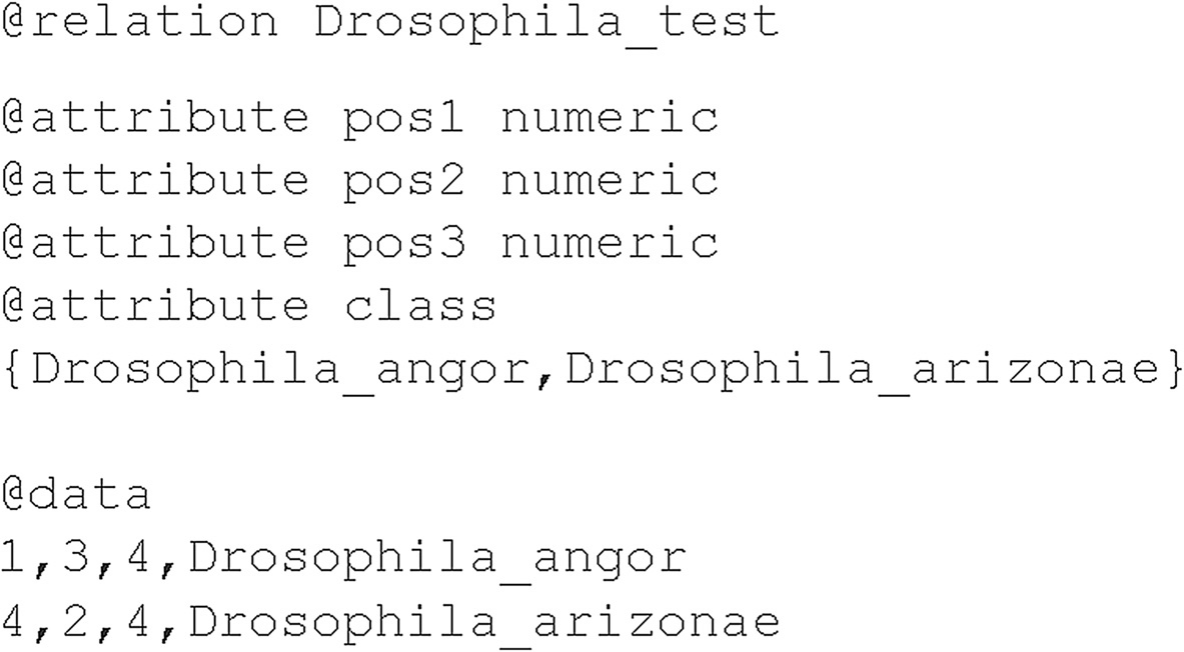
**Example of ARFF format.** The input format of the Weka package is shown: the first part of the file begins with “@relation” and includes (i) the name of the dataset and (ii) a heading line (starting with “@attribute”) for each attribute (sequence position), including the type of attribute, and the complete list of the species enclosed in braces. The second part of the file begins with “@data” and comprises a line for each specimen with the attribute values (nucleotide positions assignments) separated by comma.

In the ARFF format, the attributes represent the nucleotide positions and their assignments in the sequence, their number is equal to the sequence length plus the class label (i.e., the species). Each dataset shows the last attribute heading line (starting with “@attribute class”) comprising the species of the analyzed sequence. Moreover, the attribute values are the nucleotides (A, C, G, T) and they are mapped in a set of integer numbers from 1 to 4 (1 = A, 2 = C, 3 = G, 4 = T). Indeed, since Weka requires the same positions and the same order for categorical attributes (like A, C, G, T nucleotide assignments in the sequences) when *reference* and *query* sets are provided separately, (A, C, G, T) needed to be converted and mapped into numeric attributes (1, 2, 3, 4). In the nucleotide positions where ambiguous bases (e.g., K, M) and missing data (e.g., −) are present, the special character “?” is used for the conversion, meaning that these positions are not considered for classification purpose (i.e., only the certain bases are taken into account). An example of file in ARFF Weka format is depicted in Figure 3.

Weka supervised machine learning outputs are the classification accuracy rates of *query* and *reference* sequences, the classification models, e.g., decision trees, logic rules, etc., and the specimens to species assignments. Additional outputs can be obtained by setting specific Weka flags, see [22] and the user manual for further details.

### BLOG

Among the *ad-hoc* DNA Barcodes classification tools, a supervised machine learning method is called BLOG (Barcoding with LOGic) [7]. It is a character-based method whose aim is to classify specimens to species using classification rules that compactly characterize species in terms of DNA Barcode locations of key diagnostic nucleotides. BLOG computes for each species in the *reference* set the distinctive nucleotide positions of the DNA Barcode sequences and the logic classification rules in the form of “if-then” that are able to characterize a species in a compact way. The classification rules can then be applied to a *query* set*.* An example of classification rule is “if pos40 = T and pos265 = T then the specimen is classified as Ompok bimaculatus”. For further details on BLOG the reader may refer to [7,27,28].

### Limits of supervised methods

The following limits are identified when using supervised methods for species classification with DNA Barcode sequences:

•a full *reference* set of specimens species is necessary; at least 4 specimens per species are suggested for building a *reference* library and the sequences of each species have to include possibly all the nucleotide polymorphisms (variations); the more specimens are available, the more accurate are the classification models, and subsequently the results;

•when not using an adequate *reference* library, under-fitting or over-fitting phenomena may occur (under-fitting may be present when an insufficient number of specimens per species is given in the *reference* library, over-fitting when too many sequences of one or more species are present in the library and poor sampling is performed, i.e., not equal distributed specimens for each species);

•scaling of algorithms is not warranted when dealing with thousands of species and millions of specimens; this problem may be solved by sampling, i.e., selecting only representative sequences for each species;

•no support is provided for multi-locus DNA Barcode sequences.

## Results and discussion

### Datasets

The classification comparative analysis is performed using a selection of published empirical datasets and synthetic DNA Barcode datasets taken from [7,8,27] and available for download at dmb.iasi.cnr.it/supbarcodes.php.

### Empirical data

Public empirical datasets (available at GenBank Nucleotide Database) have been chosen with the following properties: (i) sequences with high phylogenetic diversity; (ii) identification complexity due to the absence of large inter-specific sequence differences; and (iii) selection of different genomic compartments in the sequences.

The eight selected empirical datasets, summarized in Table 3, are the following.

*Cypraeidae*[29]: *Cypraeidae* (Mollusca) are taxonomically one of the most extensively studied marine gastropods. The dataset comprises 2,008 DNA Barcode sequences with a length of 618 bases and from 211 species, where 112 species are represented by 4 or more sequences.

*Drosophila*[30]: *Drosophila* is a thoroughly studied dataset characterized by an high within-species divergence. The dataset is composed of 615 DNA Barcode sequences of 19 species; their sequence length is 663 bases and 15 species have more than five representing sequences.

*Inga*[31]: *Inga* (Fabaceae) is a large genus of tropical leguminous trees. Lots of *Inga* species collected in southwestern Amazon are sorted in an incomplete DNA Barcode tree. The dataset is made up of 913 DNA Barcodes of length 1,838. Such sequences come from 56 species, 35 are represented by more than five sequences.

*Bats*[32]: The *Bats* dataset is composed of 826 barcode sequences from specimens belonging to 82 different species. The sequences are taken from BOLD (Barcode Of Life Database) [32] and come from the Kingdom Animalia, the Phylum Chordata, the Class Mammalia, the Infraclass Eutheria, the Superorder Laurasiatheria and the Order Chiroptera.

*Fishes*[27]: The *Fishes* dataset is composed of 626 recent barcode sequences from specimens belonging to 82 different species. The Barcode sequences are obtained from GenBank Nucleotide Database and mainly taken from the Kingdom Animalia, the Phylum Chordata belonging to the commonly known paraphyletic group of the fishes.

*Birds*[33]: The *Birds* dataset is composed of 1,700 Barcode sequences from individuals that belong to 150 different species. Each fragment contains between 648 and 690 nucleotides. It was provided by the *CBOL* in the 2007 Conference (http://dimacs.rutgers.edu/Workshops/BarcodeResearchChallenges2007).

*Fungi*[4]: The *Fungi* dataset is composed of 50 sequences belonging to 8 different species. The Barcode sequences are taken from the BOLD system [32] and come from the Dikarya subkingdom.

*Algae*[3]: The *Algae* dataset is composed of 26 sequences belonging to 8 different species. The Barcode sequences are taken from the BOLD system [32] and come from the Haematococcaceae family of green algae.

**Table 3:** Summary of the empirical datasets

**Dataset**	**#sequences**	**Seq. length**	**#species**	**Gene region(s)**	**Ref**
**Cypraeidae**	2,008	614	211	*COI*	[29]
**Drosophila**	615	663	19	*COI*	[30]
**Inga**	913	1,838	56	*tmTD, ITS*	[31]
**Bats**	826	659	82	*COI*	[32]
**Fishes**	626	419	82	*COI*	[27]
**Birds**	1,700	255	150	*COI*	[33]
**Fungi**	50	510	8	*ITS*	[4]
**Algae**	26	1,128	5	*rbcL*	[3]

Legend: #Sequences = number of dataset sequences comprised in the dataset; Seq. length = length of the sequences; #Species = number of species in the datasets; Gene Region(s) = gene region(s) used as Barcode for each dataset; Ref = reference to original publication.

### Synthetic data

Real DNA Barcode datasets are simulated with Coalescent package in Mesquite version 2.75 (see the related work [8]). The data are simulated considering time of species divergence and the effective population size (*Ne*), i.e., the number of individuals in a population (of a species) that are contributing genes to the succeeding generations. Firstly, according to the Yule coalescence model [8], gene trees with 1,000, 10,000, and 50,000 individuals of effective population size are simulated, generating datasets composed of 50 species each of 20 individuals (Table 4). Each simulation is replicated in a 100-fold scheme. The dataset complexity increases with population size. Then, DNA Barcode sequences are simulated on the addictive gene trees, with a sequence length of 650 bases, similar to the real size of a standard DNA Barcode.

**Table 4:** Summary of the synthetic datasets

**Dataset**	**Ne**	**#individual**	**Seq. length**	**#species**	**Ref**
**Ne1000**	1,000	20	650	50	[8]
**Ne10000**	10,000	20	650	50	[8]
**Ne50000**	50,000	20	650	50	[8]

Legend: #Individual = number of sequences for each species; Seq. length = length of the sequences; #Species = number of species in the datasets; Ref = reference to original publication.

### Data sampling

The sequences of the empirical selected datasets are divided into a *reference* set (80% per species), including the sequences with *a priori* assigned species membership, and a *query* set (20% per species), comprising also the DNA Barcode sequences with an *a priori* assigned species label (but not considered by the algorithm) for an evaluation of the classification success. Also the synthetic DNA Barcode sequences are divided into *reference* dataset and *query* dataset, which include 16 and 4 sequences for species, respectively. It is worth noting that since species membership of *query* dataset is simulated together with the *reference* dataset, they are also known, allowing *a posteriori* evaluation of their identification accuracy.

The samplings, i.e., the divisions of *reference* and *query* set, are performed according to the same data splits present in previous works [7,8,27] for allowing a comparison of the classification results. These data splits were performed by biologists in [8], following specific sequence compositions (e.g., polymorphism) and challenges (e.g., low species divergences, not equal-distributed specimen for each species, and high intra-species variability). Moreover, when possible each dataset is composed of species with 5 or more representing sequences in the *reference* library.

### Experimental settings

A typical experimentation procedure is described in this section. Moreover, a comprehensive tutorial that guides the user during the software package downloads, set up, and the execution of the experiments on its own datasets is provided as Additional file 1.

The supervised machine learning classification analysis of the eight selected empirical datasets (*Cypraeidae*, *Drosophila*, *Inga*, *Bats*, *Fishes*, *Birds*, *Fungi*, *Algae*) is performed according to the following steps:

1. the sequences are acquired from dmb.iasi.cnr.it/supbarcodes.php;

2. each dataset (*reference* and *query*) is converted in Weka ARFF format with the special converter described previously in the *Input, sequences conversion and output* section;

3. the supervised machine learning algorithms C4.5, Naïve Bayes, RIPPER, and SVM are run in Weka;

4. the specimen to species classification accuracies and the classification models are evaluated.

The analysis of the selected synthetic datasets (Ne1000, Ne10000, Ne100000) is performed according to the following steps:

1. the sequences are acquired from dmb.iasi.cnr.it/supbarcodes.php;

2. each dataset (*reference* and *query*) is converted in Weka ARFF format with the special converter described previously in *Input, sequences conversion and output* section;

3. the supervised machine learning algorithms C4.5, Naïve Bayes, RIPPER, and SVM are run in Weka 100 times on different *reference* – *query* splits; special scripts for performing a batch classification analysis in Weka have been implemented and are available upon request;

4. the specimen to species classification accuracies and the classification models are evaluated;

5. the average classification accuracies of the 100 runs are computed.

Moreover, the Multi-Layer Perceptron method [34] has been tested, however it required a very high running time, not providing the demanded output even after hours of computation. Therefore, the results have been not considered in the comparison.

To evaluate the performances of the algorithms, accuracy and standard deviation, both weighted by the number of samples for each dataset, are considered. In addition, as statistical test of differences among algorithms, the pairwise Wilcoxon signed rank test based on paired observations [35] has been performed.

### Parameter configurations

The supervised classification algorithms are tested using both the standard configuration and a comprehensive parameter tuning (see the following *Comparative Analysis* subsection for the obtained results). Specifically, the standard parameters for each analyzed method are listed in Additional file 2: Table S1.

### Empirical sequences: classification analysis and results

Eight empirical DNA Barcode sequence datasets have been analyzed for classification according to the steps described in the previous section.

The accuracies on the *query* set of all empirical datasets are listed in Table 5, as well as the averaged accuracy with its standard deviation, both weighted by the number of samples for each dataset. SVM and Naïve Bayes reach the highest classification performances on all tested datasets. As expected, the statistical difference between SVM and Naïve Bayes resulted not significant (p-value > 0.05) according to the pairwise Wilcoxon test. On the other hand, the observed differences computed among SVM (Naïve Bayes) and the other algorithms resulted statistically significant (p-value ≤ 0.001).

**Table 5:** Accuracies for the empirical datasets [%]

**Dataset**	**SVM**	**Jrip**	**J48**	**Naïve Bayes**	**Average**	**Standard deviation**
**Cypraeidae**	**94.32**	86.93	91.76	93.18	91.55	2.82
**Drosophila**	**98.28**	94.83	91.38	96.55	95.26	2.55
**Inga**	89.83	88.14	88.14	**91.53**	89.41	1.41
**Bats**	**100.00**	**100.00**	98.15	**100.00**	99.54	0.80
**Fishes**	95.50	90.09	92.79	**97.30**	93.92	2.73
**Birds**	**98.42**	84.86	91.80	94.32	92.35	4.93
**Fungi**	**80.00**	50.00	60.00	70.00	65.00	11.20
**Algae**	**100.00**	60.00	60.00	**100.00**	80.00	20.00

Results of the Weka supervised learning methods tested on empirical datasets show that SVM and Naïve Bayes outperform the other techniques in term of percentage of the correct species identification. The differences between SVM and the other algorithms result statistically significant (p-value ≤ 0.001), except for Naive Bayes (p-value > 0.05). The best performances are highlighted in bold for each dataset.

The detailed results of the supervised machine learning tested methods are shown for the eight empirical datasets and the performances on *query* set and *reference* set for each selected empirical dataset are drawn in Additional file 2: Figures S1-S8. Each figure depicts results on empirical data through histograms that provide the accuracy rate for all analyzed methods on the *query* set (panel (a) of each picture) and on the *reference* set (panel (b) of each picture).

### Synthetic sequences: classification analysis and results

Three synthetic DNA Barcode sequence datasets have been analyzed for the classification according to the steps described in section *Experimental settings*.

The classification performances on *query* and *reference* sets of synthetic datasets with *Ne* equal to 1,000, 10,000, and 50,000 are summarized in Table 6. The weighted average accuracy on the *query* set is around 96% for both *Ne* equal to 1,000 and 10,000, and 91% for *Ne* equal to 50,000 (Table 6).

**Table 6:** Accuracies for the synthetic datasets [%]

**Dataset**	**SVM**	**Jrip**	**J48**	**Naïve Bayes**	**Average**	**Standard deviation**
**Ne1000**	**96.53**	96.26	94.07	96.48	95.84	1.19
**Ne10000**	96.77	95.26	94.88	**96.79**	95.93	0.99
**Ne50000**	**93.92**	89.28	89.63	92.46	91.32	2.24

Results of the Weka supervised learning methods tested on synthetic datasets show that SVM and Naïve Bayes outperform the other techniques in term of percentage of the correct species identification. The differences between SVM and the other algorithms result statistically significant (p-value ≤ 0.001). The best performances are highlighted in bold for each dataset.

The results on the synthetic data are largely consistent with results on the empirical ones: SVM and Naïve Bayes outperform the other methods. The statistical significance (p-value ≤ 0.001) is proven by performing the pairwise Wilcoxon test among SVM (Naïve Bayes) and the other algorithms with a Bonferroni correction [36] in order to consider the high numbers of comparisons. In this case, also the performance difference between SVM and Naïve Bayes is statistically significant (p-value ≤ 0.001).

The detailed performances are reported in Additional file 2: Figure S9, S10 and S11. Each figure depicts results on synthetic data through histograms and bar-plots, in order to highlight the averaged performances (panels (b) and (d) of each picture) together with the standard deviation (panels (a) and (c) of each picture).

### Comparative analysis

A comparative evaluation of the classification results is performed (i) using several machine learning algorithms from the collection of Weka classifiers; (ii) using these algorithms with different parameter configurations; and (iii) comparing the results with *ad-hoc* and well-established DNA Barcode classification techniques, as phylogenetic trees (NJ, PAR), similarity-based (BLAST), and character-based (DNA-BAR, BLOG) methods. The results are compared evaluating accuracy and standard deviation, both weighted by the number of samples for each dataset.

### Supervised machine learning algorithm comparisons

The different Weka supervised machine learning algorithms are run on empirical and synthetic data according to the steps previously described in section *Experimental setting.*

**Figure 4:**
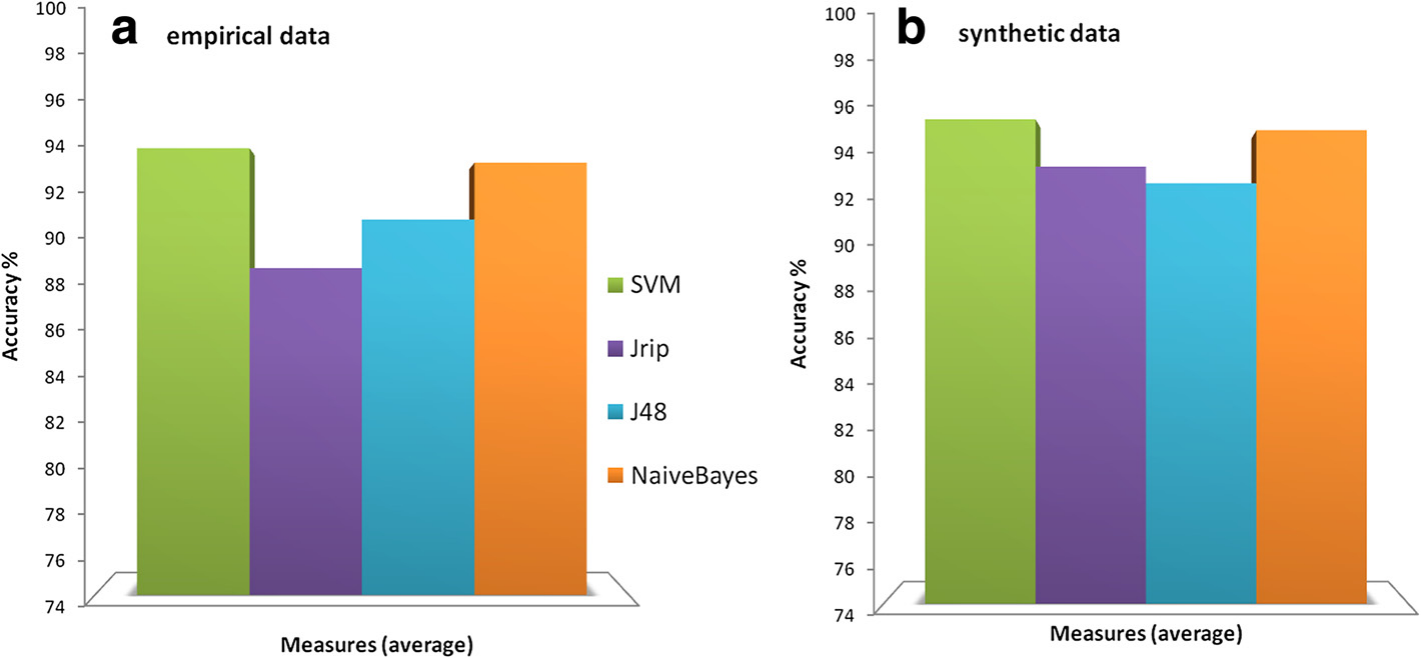
**Weka supervised machine learning methods comparison. (a)** The DNA Barcode *query* identification success scores of SVM, Jrip, J48, Naïve Bayes tested on the eight empirical datasets are depicted; **(b)** The DNA Barcode *query* identification success scores of SVM, Jrip, J48, Naïve Bayes tested on the three synthetic datasets are depicted.

The comparative evaluation of Weka classifiers shows that SVM and Naïve Bayes methods outperform on average the other classifiers (Jrip, J48), both on empirical (panel (a) of Figure 4) and synthetic (panel (b) of Figure 4) datasets, although a precise and human interpretable classification model is not provided, as the one of rule-based methods (e.g., Jrip). Note that the performance differences are statistically significant, as explained in subsections *Empirical (Synthetic) sequences: classification analysis and results*.

### Default *versus* different parameter configurations of Weka classifiers

Different parameter settings of the supervised machine learning algorithms in Weka have been tested on empirical data according to the steps described in section *Experimental settings*. The standard classification performances of machine learning methods on three selected empirical datasets (i.e., *Cypraeidae*, *Drosophila* and *Inga*) are compared with respect to the ones obtained using other parameter configurations (listed in Additional file 2: Table S2, S3, S4 for *Cypraeidae*, *Drosophila* and *Inga*, respectively). The results of the comparative analysis for the three empirical datasets are shown in Additional file 2: Figure S12-S14. No relevant differences among the analyzed configurations appear, except for the configuration of *Drosophila* and *Inga* when SVM uses a Logistic Model. Only three datasets are taken as representative samples and analyzed using different parameters, as the classification results do not substantially change when performing parameters tuning.

### Weka algorithms *versus* DNA Barcodes *ad-hoc* classification methods

In this experimentation the empirical and synthetic datasets (*Cypraeidae*, *Drosophila*, and *Inga*) have been analyzed with Weka supervised machine learning algorithms according to the steps described in section *Experimental settings* and their accuracy has been compared to previous results presented in [8].

**Figure 5:**
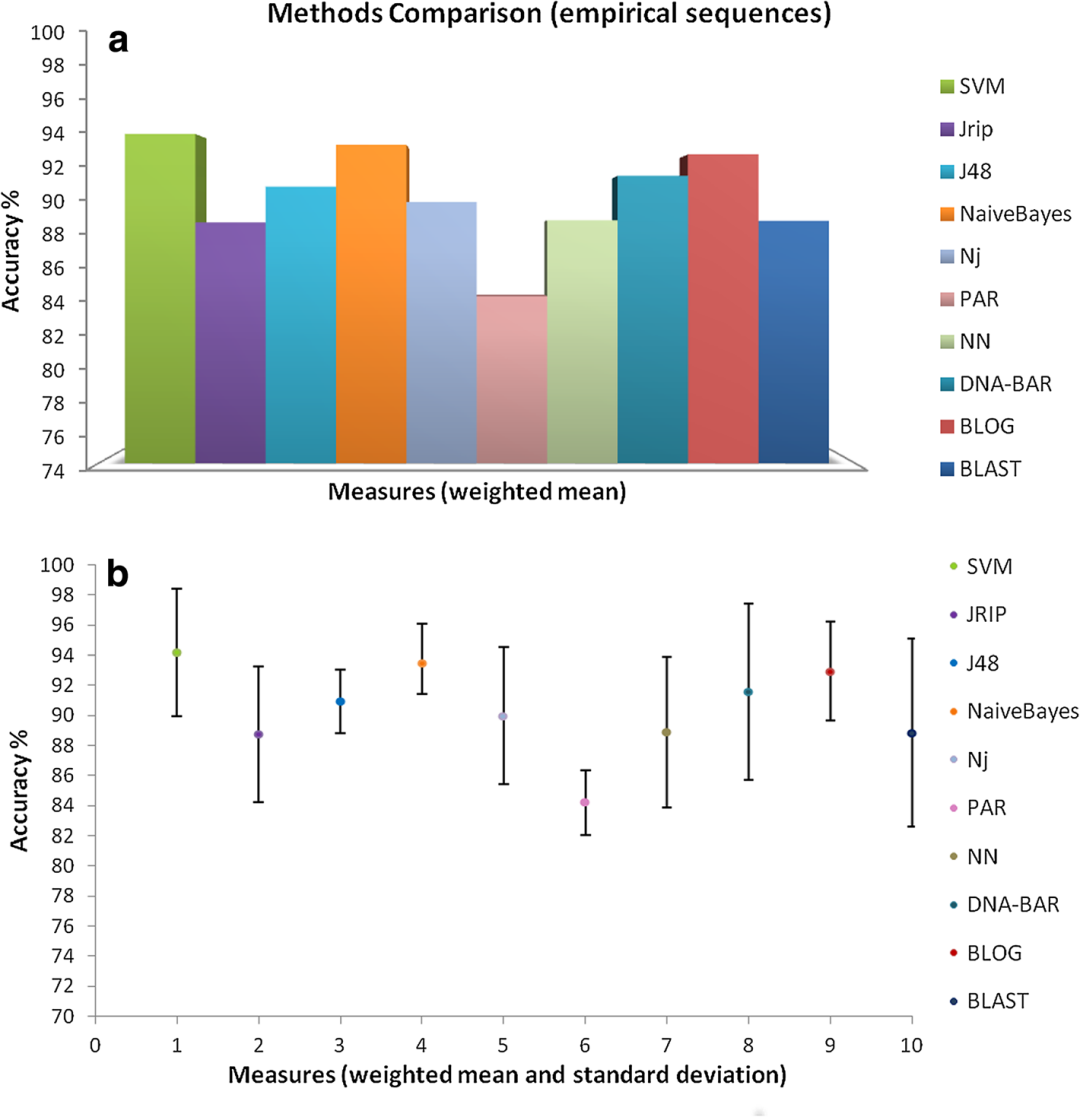
**Classification performances (accuracy) for the empirical datasets. (a)** DNA Barcode *query* identification success scores of Weka methods (SVM, Jrip, J48, Naïve Bayes) together with other *ad-hoc* methods for DNA Barcode analysis (NJ, PAR, NN, DNA-BAR, BLAST, BLOG) applied to three empirical datasets (i.e., *Cypraeidae*, *Inga*, and *Drosophila*) are depicted; **(b)** bar-plot of DNA Barcode *query* identification success scores: the weighted accuracy of each method averaged over the samples of the three empirical datasets and their standard deviations are depicted.

**Figure 6:**
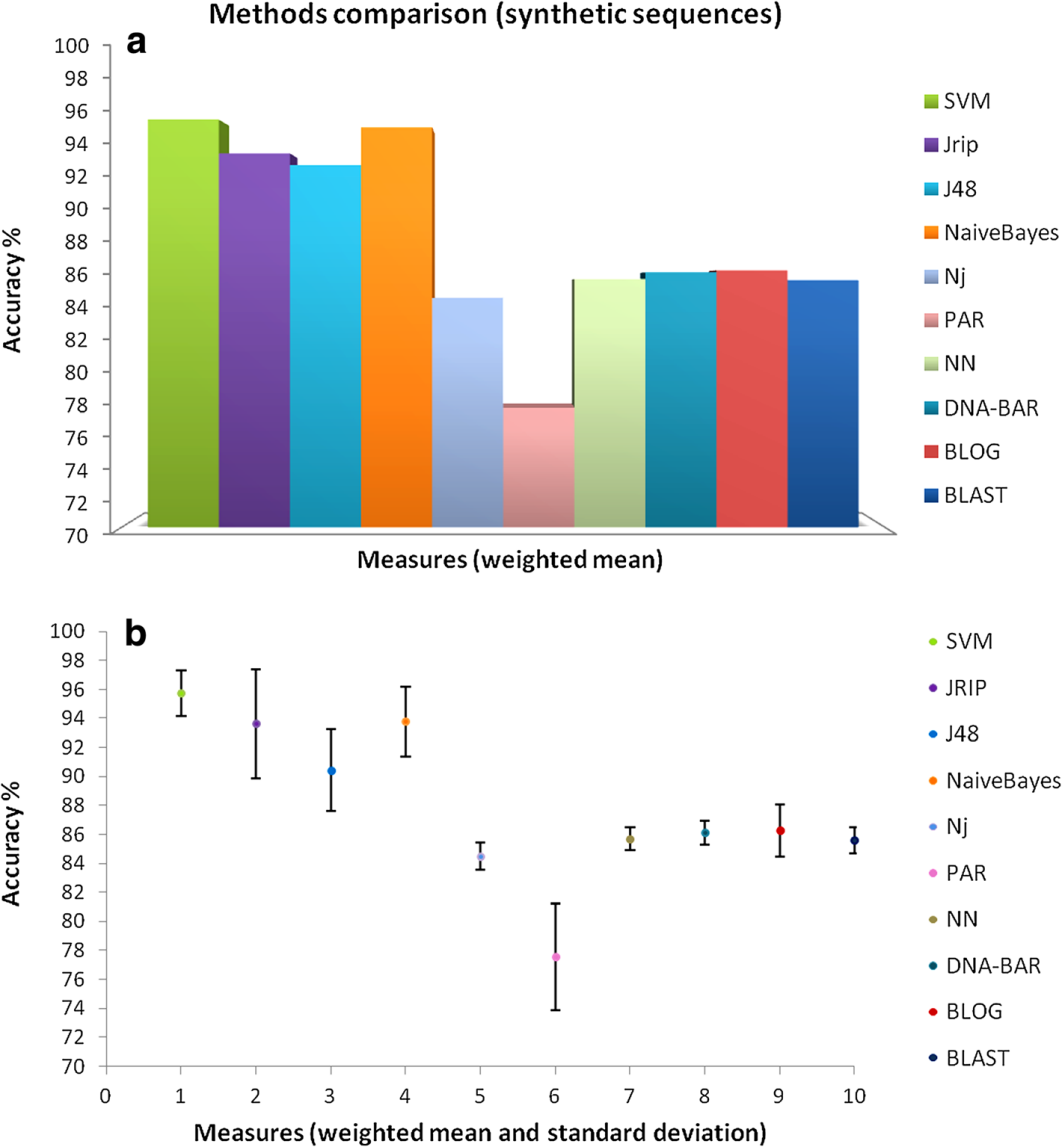
**Classification performances (accuracy) for the synthetic datasets. (a)** The DNA Barcode *query* identification success scores of Weka methods (SVM, Jrip, J48, Naïve Bayes) together with *ad-hoc* DNA Barcode analysis methods (NJ, PAR, NN, DNA-BAR, BLAST, BLOG) applied to the three synthetic Barcode sequence datasets (i.e., Ne1000, Ne10000, Ne50000) are depicted; **(b)** bar-plot of the DNA Barcode *query* identification success scores: the weighted accuracy of each method averaged over the samples of the three synthetic datasets and their standard deviations are depicted.

Analysis results on empirical (Figure 5) and synthetic (Figure 6) datasets show that two Weka classifiers (Naïve Bayes and SVM) reach on average the highest classification performances with respect to the other *ad-hoc* DNA Barcode analysis methods (although note that not all of them are statistically significant according to the Wilcoxon test). However, Naïve Bayes and SVM do not provide a clear and compact human interpretable classification model. Rule-based methods [37], as BLOG [7] and RIPPER [24], have lower classification performances, but the user is provided with the diagnostic positions and the nucleotide assignments (e.g., “if pos40 = T and pos265 = T then the specimen is classified as Ompok bimaculatus”). It is worth nothing that the differences between SVM performances and character-based methods (DNA-BAR and BLOG) are not statistically significant (p-value > 0.05).

Summarizing, on synthetic data the supervised machine learning methods outperform the *ad-hoc* DNA Barcode classification methods (Figure 6), although not all of them results statistically significant according to the Wilcoxon test. On empirical data the classification performances are comparable to the *ad-hoc* methods (Figure 5). The empirical datasets taken into account for this comparison are only the *Cypraeidae*, *Drosophila*, and *Inga* sequences, as tested in previous studies [8]. It is not surprising that *ad-hoc* DNA Barcodes classification methods have slightly weaker performances on synthetic data, as the sequences are generated to challenge these methods.

## Conclusions

This paper provides a comprehensive approach to the problem of assigning an unknown specimen to a known species by analyzing its DNA Barcode. Such a task was addressed using supervised classification algorithms implemented by the software tool Weka. In particular, specific classifiers like the function-based method Support Vector Machines (SVM), the rule-based RIPPER (Jrip), the decision tree C4.5, and the Bayesian-based method Naïve Bayes were tested on synthetic and empirical datasets belonging to the animal, fungus, and plant kingdoms. Additionally, an integrated tool that converts the DNA Barcode FASTA sequences to the Weka format was developed in order to adapt different input formats and hence to allow the experiments execution.

Furthermore, the classification results were compared with respect to *ad-hoc* and well-established DNA Barcode classification techniques, as phylogenetic trees (NJ, PAR), similarity-based (BLAST), and character-based (DNA-BAR, BLOG) methods. The classification analysis shows that supervised machine learning methods are promising candidates for handling with success the DNA Barcode species classification problem, obtaining excellent classification performances. On empirical data the classification performances were comparable to the traditional DNA Barcode classification methods, while on synthetic data higher classification performances have been obtained. The results presented in this paper and those available in previous literature establish the extensive validity of the application of supervised learning methods for species classification with DNA Barcodes, testing both the accuracy of different methods and of different dataset types. Finally, a powerful tool and pipeline to perform species classification are provided to the DNA Barcoding community.

An extension of the supervised classification procedure is planned as future work, where the issue of specimen to species assignments with multi-locus DNA Barcode sequences will be analyzed and addressed.

## Competing interests

The authors declare that they have no competing interests.

## Authors’ contributions

EW conceived the idea, designed the analysis method, and developed the FASTA to Weka converter. GiuFis collected and analyzed the data. EW and GiuFis interpreted the data, the results, and wrote the paper. GioFel directed research. All authors read and approved the final manuscript.

## Supplementary Material

Additional file 1 Provides a tutorial that guides the user during the software package downloads, set up, and the execution of the experiments on its own datasets. Click here for file

Additional file 2 Additional results and tables of the classification analysis on empirical and synthetic datasets. Click here for file
