# Supplementary material for: Supervised DNA Barcodes species classification: analysis, comparisons and results
Source: BioData Min. 2014 Apr 11;7:4. doi: 10.1186/1756-0381-7-4 (PMC4022351; doi:10.1186/1756-0381-7-4)
Supplement: Additional file 1 — Provides a tutorial that guides the user during the software package downloads, set up, and the execution of the experiments on its own datasets. [file 1756-0381-7-4-S1.pdf]

Supplementary material for the paper:

SUPERVISED DNA BARCODES SPECIES CLASSIFICATION: ANALYSIS, COMPARISON AND RESULTS

Emanuel Weitschek, Giulia Fiscon, Giovanni Felici

Parameters configuration

Table S1 lists the standard configuration of parameters set to test the performances of the selected classification algorithms of Weka package (SVM, Jrip, J48, Naïve Bayes).

Table S1 Configuration Parameters

Classifier	Parameters	Description	Value
**SMO (SVM)**	build logistic models	whether to fit logistic models to the outputs	FALSE
	c	complexity parameter C	1.0
	epsilon	epsilon for round-off error	10^{-12}
	filterType	determines how/if the data will be transformed	normalized
	kernel	kernel to use	polyKernel
	numFolds	number of folds for cross-validation used to generate training data for logistic models	-1
	tolerance parameters	tolerance parameter	0.001
	random seed	number seed for cross-validation	1
**Jrip**	folds	amount of data used for pruning. (one fold is used for pruning, the rest for growing the rules)	3
	seed	seed used for randomizing the data	1
	minNO	minimum total weight of the instances in a rule	2.0
	optimizations	number of optimization runs	2
	use pruning	whether pruning is performed	TRUE
**J48**	confidence factor	confidence factor used for pruning	0.25
	minNumOb	minimum number of instances per leaf	2
	numFolds	number of folds for cross-validation used to generate training data for logistic models	3
	reduced-error pruning	whether reduced-error pruning is used instead of C.4.5 pruning	FALSE
	sub tree raising	whether to consider the subtree raising operation when pruning	TRUE
	seed	seed used for randomizing the data when reduced-error pruning	1
	unpruned	whether pruning is performed	FALSE
	use Laplace	whether counts at leaves are smoothed based on Laplace	FALSE
**Naïve Bayes**	supervised discretization	use supervised discretization to convert numeric attributes to nominal ones	FALSE
	kernel estimator	use a kernel estimator for numeric attributes rather than a normal distribution	FALSE

Empirical sequences classification results

The results of the supervised machine learning tested methods are shown for the eight empirical datasets (Cypraiede, Drosophila, Inga, Bats, Fishes, Birds, Fungi, Algae). In particular, the performances on query set (test set) and reference set (training set) for each selected empirical dataset are drawn in Figure S1-S8. Each figure depicts results on empirical data through histograms that provide the accuracy rate for all analyzed methods on the query set (panel **(a)** of each picture) and reference set (panel **(b)** of each picture).

Cypraeidae

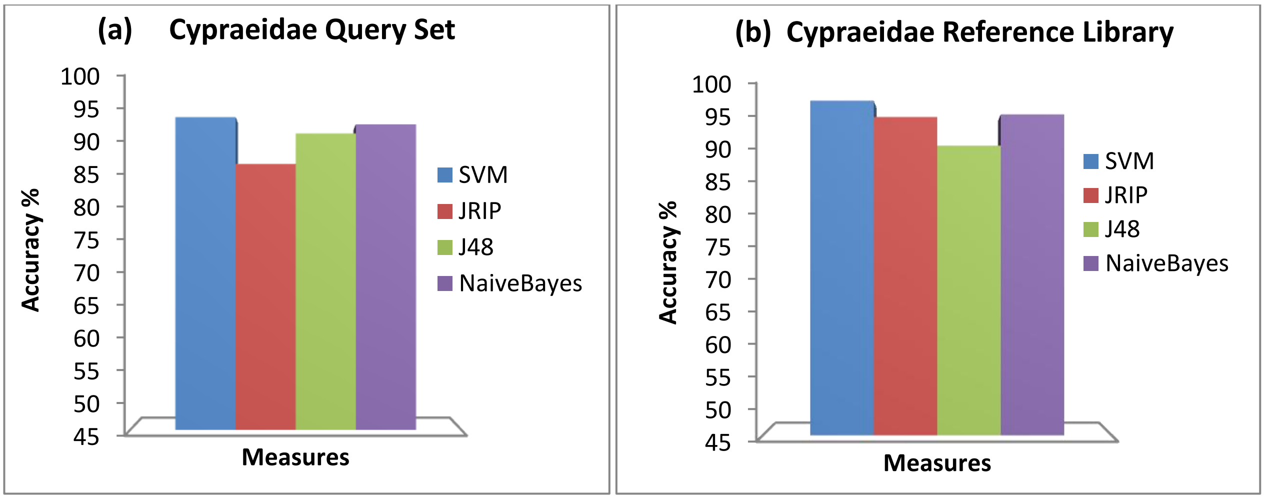

Figure S1 Results on Cypraeidae dataset: (a) DNA Barcode query identification success scores of four methods applied to Barcode sequence dataset; (b) DNA Barcode reference success scores of four methods applied to Barcode sequence dataset.

Drosophila

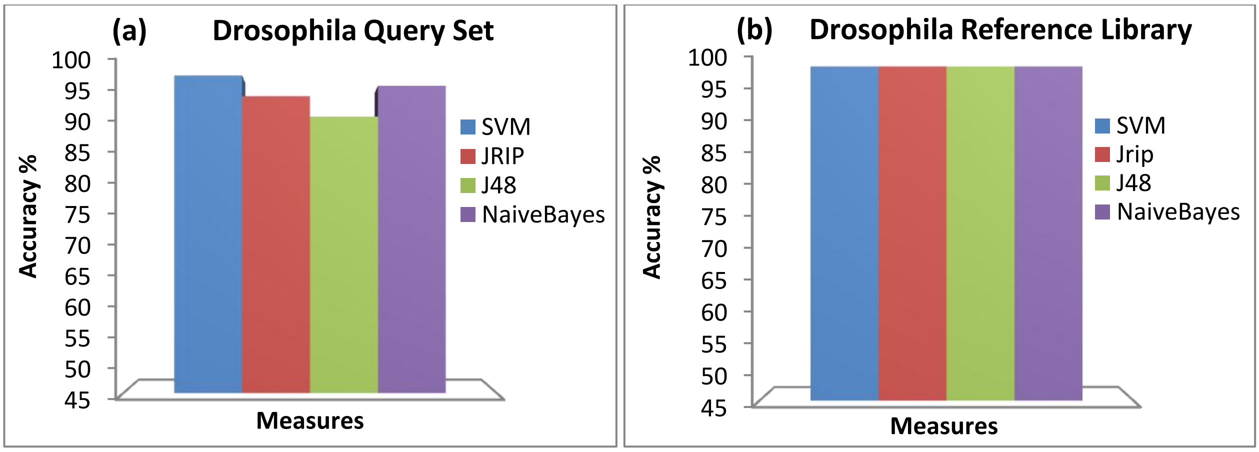

Figure S2 Results on Drosophila dataset: (a) DNA Barcode query identification success scores of four methods applied to Barcode sequence dataset; (b) DNA Barcode reference success scores of four methods applied to Barcode sequence dataset.

Inga

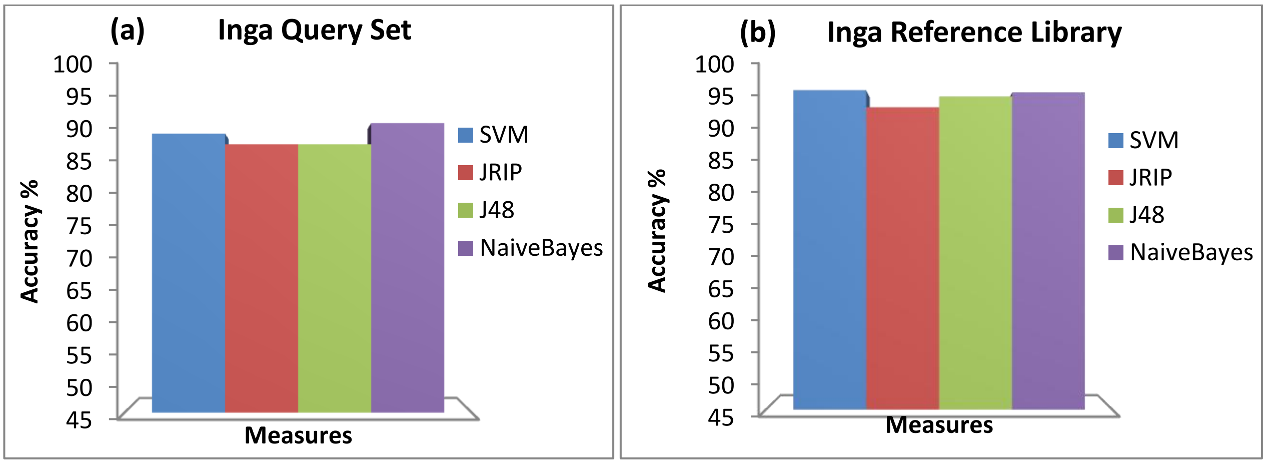

Figure S3 Results on Inga dataset: (a) DNA Barcode query identification success scores of four methods applied to Barcode sequence dataset; (b) DNA Barcode reference success scores of four methods applied to Barcode sequence dataset.

Bats

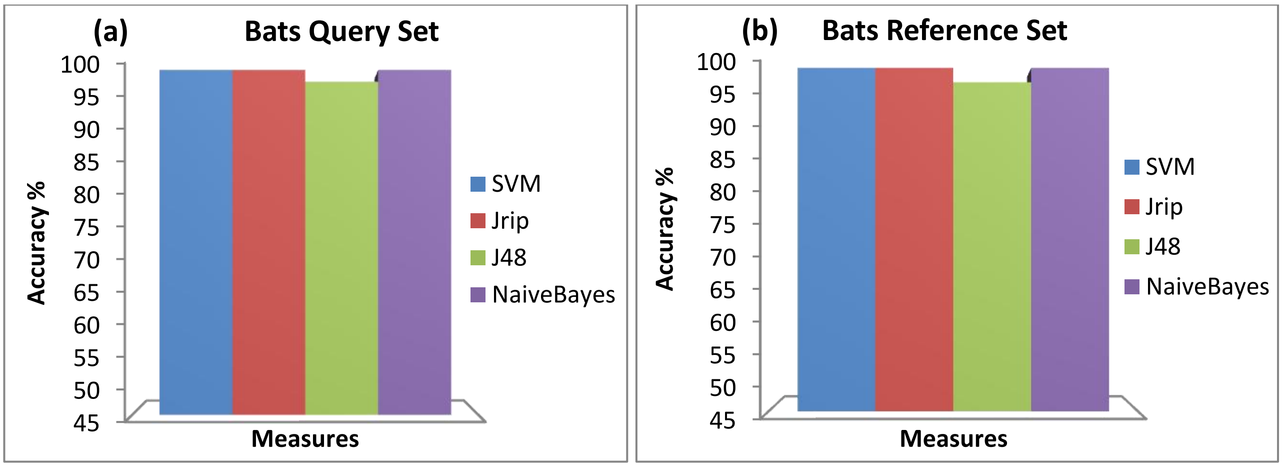

Figure S4 Results on Bats dataset: (a) DNA Barcode query identification success scores of four methods applied to Barcode sequence dataset; (b) DNA Barcode reference success scores of four methods applied to Barcode sequence dataset.

Fishes

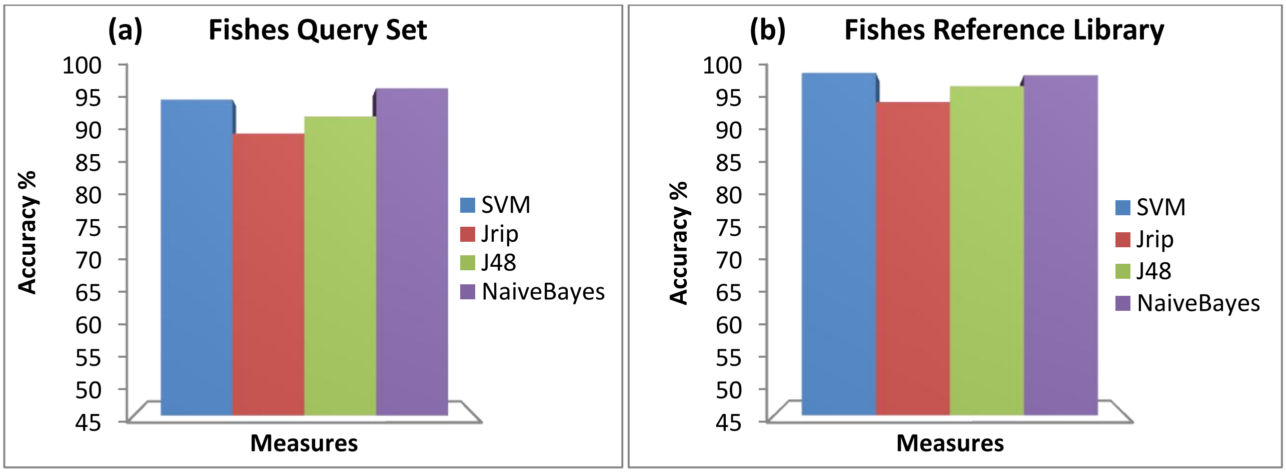

Figure S5 Results on Fishes dataset: (a) DNA Barcode query identification success scores of four methods applied to Barcode sequence dataset; (b) DNA Barcode reference success scores of four methods applied to Barcode sequence dataset.

Birds

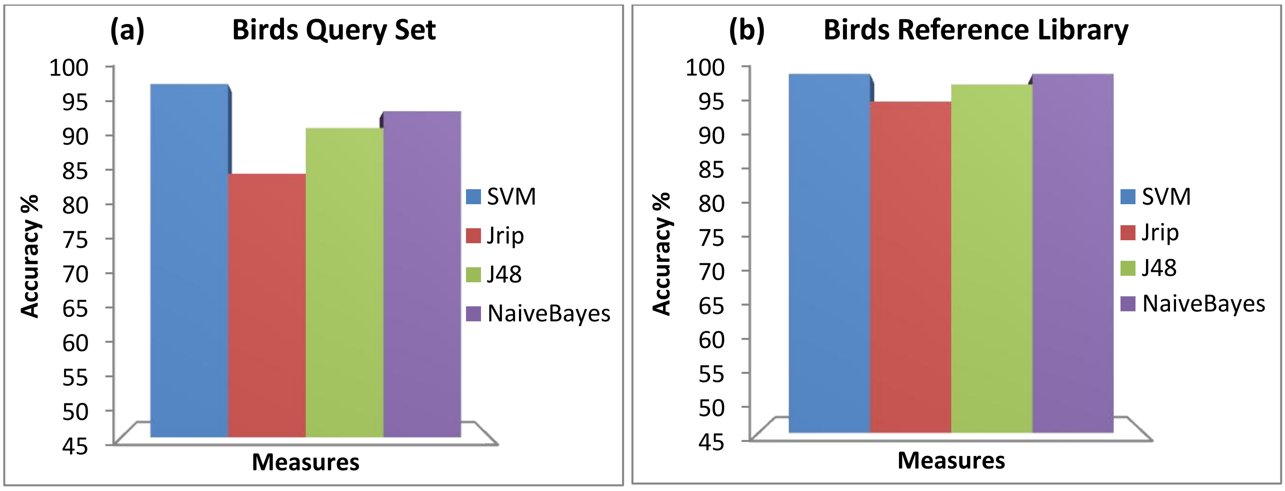

Figure S6 Results on Birds dataset: (a) DNA Barcode query identification success scores of four methods applied to Barcode sequence dataset; (b) DNA Barcode reference success scores of four methods applied to Barcode sequence dataset.

Fungi

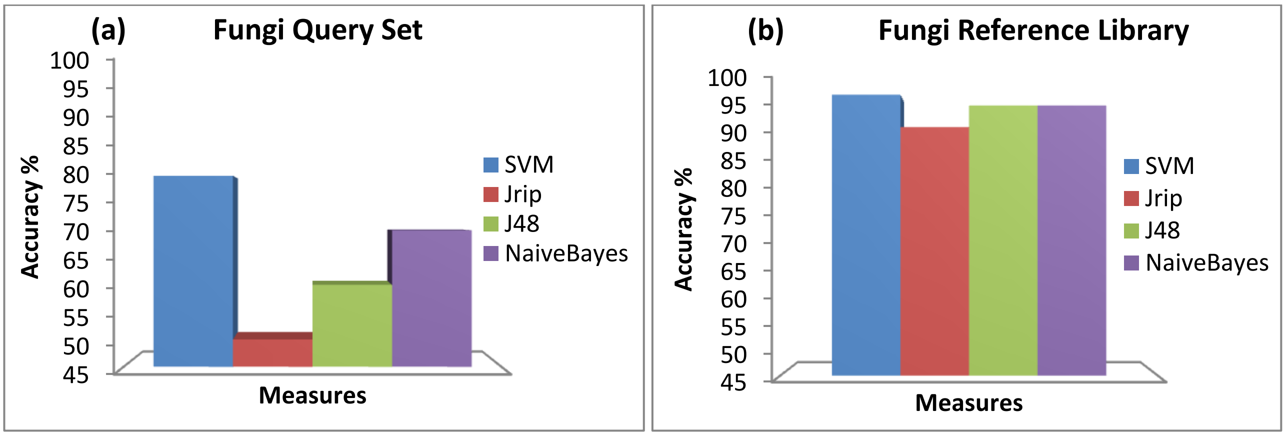

Figure S7 Results on Fungi dataset: (a) DNA Barcode query identification success scores of four methods applied to Barcode sequence dataset; (b) DNA Barcode reference success scores of four methods applied to Barcode sequence dataset.

Algae

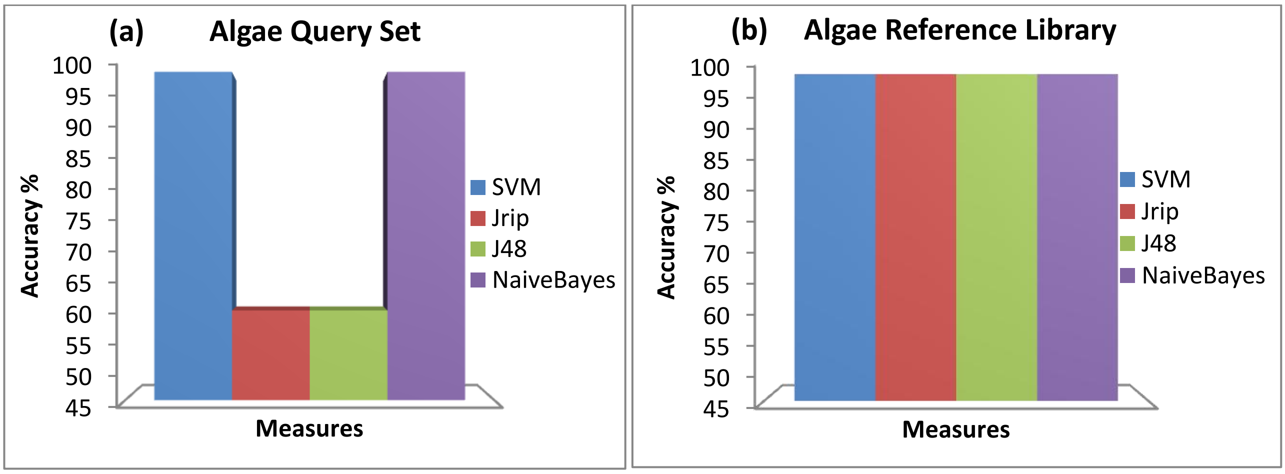

Figure S8 Results on Algae dataset: (a) DNA Barcode query identification success scores of four methods applied to Barcode sequence dataset; (b) DNA Barcode reference success scores of four methods applied to Barcode sequence dataset.

Synthetic sequences classification results

The classification performances on query (test) and reference (training) sets of synthetic data with *Ne* equal to 1,000 – 10,000 – 50,000 are shown in Figure S9, Figure S10 and Figure S11, respectively. Each figure depicts results on synthetic data through histograms and bar-plots, in order to highlight the averaged performances (panels **(b)** and **(d)** of each picture) together with the standard deviations (panels **(a)** and **(c)** of each picture).

Ne 1000

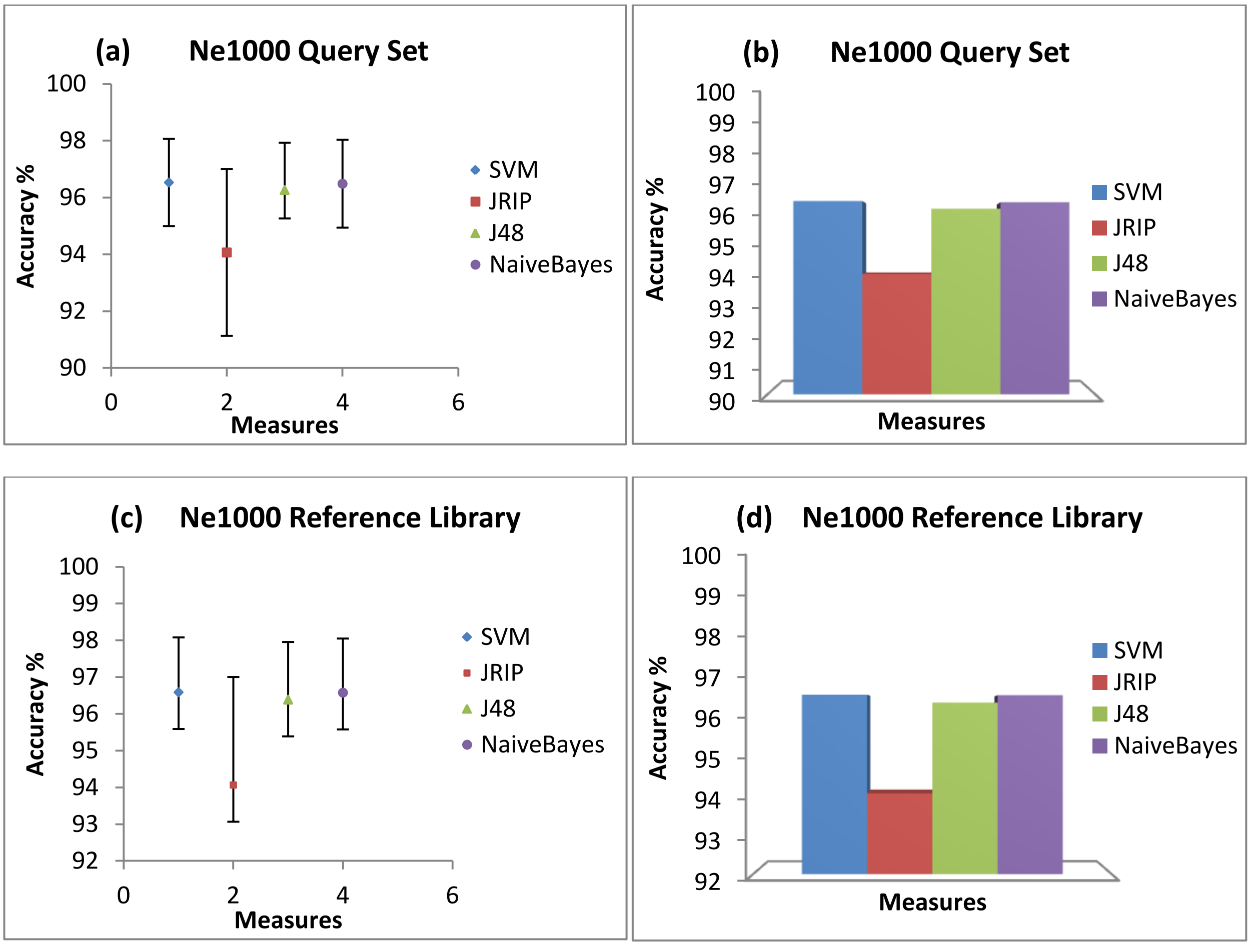

Figure S9 Results of synthetic dataset with *Ne* equal to 1000: (a) bar-plot of DNA Barcode query identification success scores considering for each method the averaged accuracy and its standard deviation; (b) DNA Barcode query identification success scores of four methods applied to Barcode sequence dataset; (c) bar-plot of DNA Barcode reference success scores considering for each method the averaged accuracy and its standard deviation; (d) DNA Barcode reference success scores of four methods applied to Barcode sequence dataset.

Ne 10000

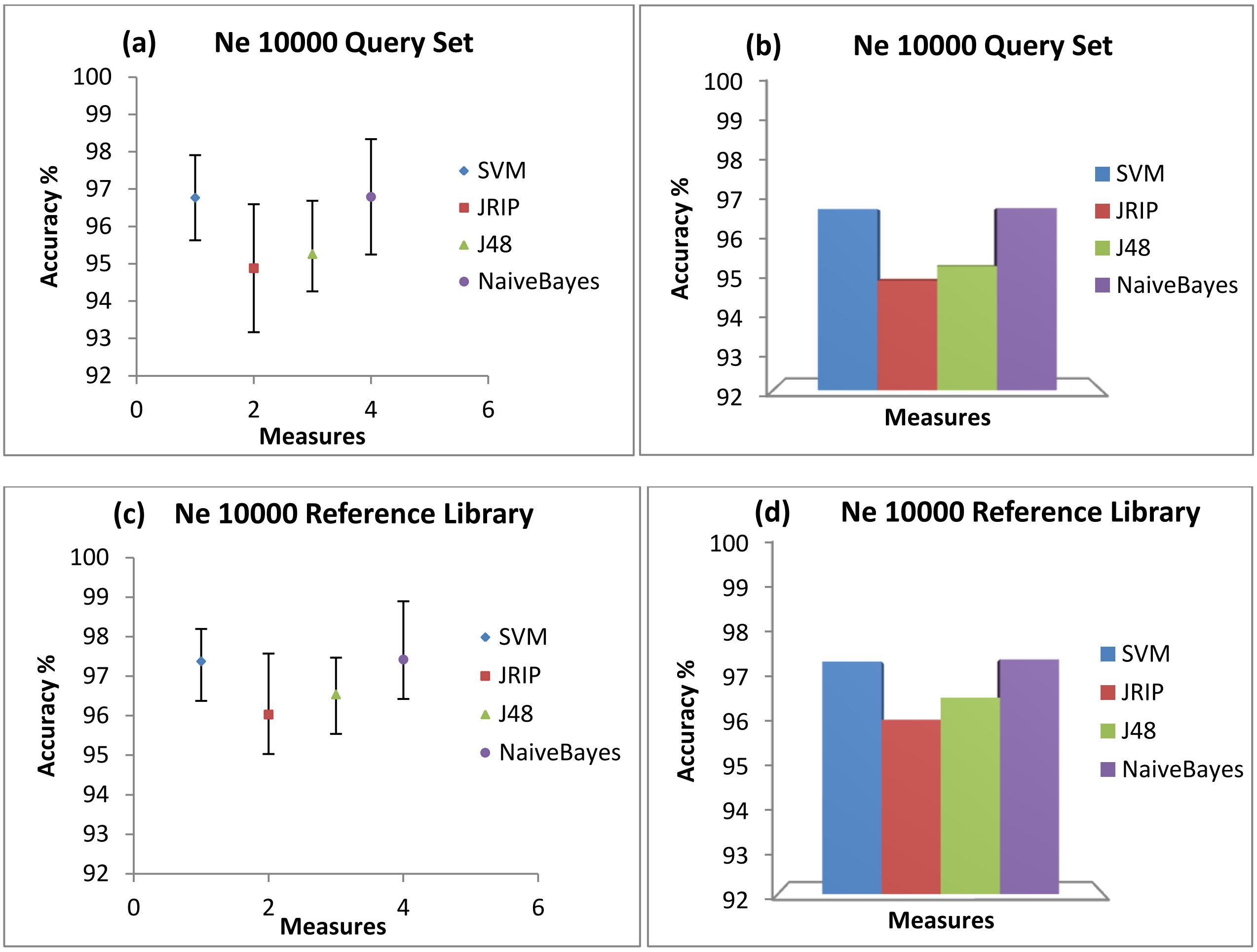

Figure S10 Results of synthetic dataset with *Ne* equal to 10000: (a) bar-plot of DNA Barcode query identification success scores considering for each method the averaged accuracy and its standard deviation; (b) DNA Barcode query identification success scores of four methods applied to Barcode sequence dataset; (c) bar-plot of DNA Barcode reference success scores considering for each method the averaged accuracy and its standard deviation; (d) DNA Barcode reference success scores of four methods applied to Barcode sequence dataset.

Ne 50000

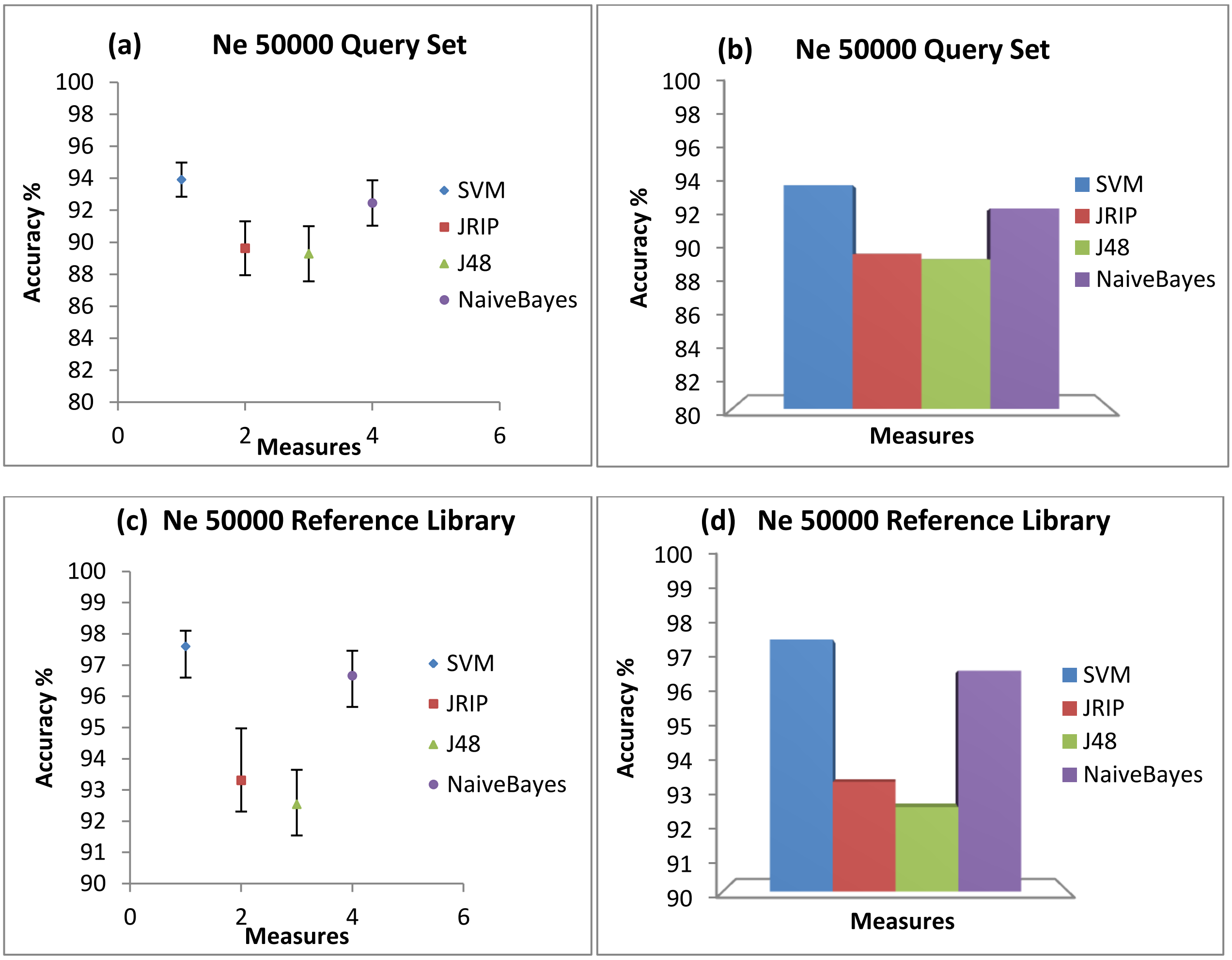

Figure S11 Results of synthetic dataset with *Ne* equal to 50000: (a) bar-plot of DNA Barcode query identification success scores considering for each method the averaged accuracy and its standard deviation; (b) DNA Barcode query identification success scores of four methods applied to Barcode sequence dataset; (c) bar-plot of DNA Barcode reference success scores considering for each method the averaged accuracy and its standard deviation; (d) DNA Barcode reference success scores of four methods applied to Barcode sequence dataset.

Default VS different parameter configurations of Weka classifiers

Diverse parameter settings of the supervised machine learning algorithms in Weka have been tested on empirical and synthetic data according to the steps described in section *Experimental settings* of the paper. The classification performances of machine learning methods on three selected empirical datasets (i.e., Cypraeidae, Drosophila and Inga), using the configuration parameters of Table S1, are compared with the ones obtained using other configurations, that are listed in Table S2-S4 for Cypraeidae, Drosophila and Inga, respectively. The results of comparative analysis for the three empirical data sets are depicted in Figure S9-S11. No meaning differences among the analyzed configurations does exist, except to the configuration of Drosophila and Inga (number 3 in Table S3 and Table S4), where SVM uses a Logistic Model.

Cypraeidae

Table S2 Cypraeidae parameters configuration (see Table 3 for explanation of the default parameters configuration)

#Configuration	Jrip	SVM	J48	Naïve Bayes
1	default	default	default	default
2	pruning=FALSE	built logistic model	reduce error pruning =TRUE	kernel estimator=TRUE
3	optimization=4	-	unpruned = TRUE	supervised discretization=TRUE

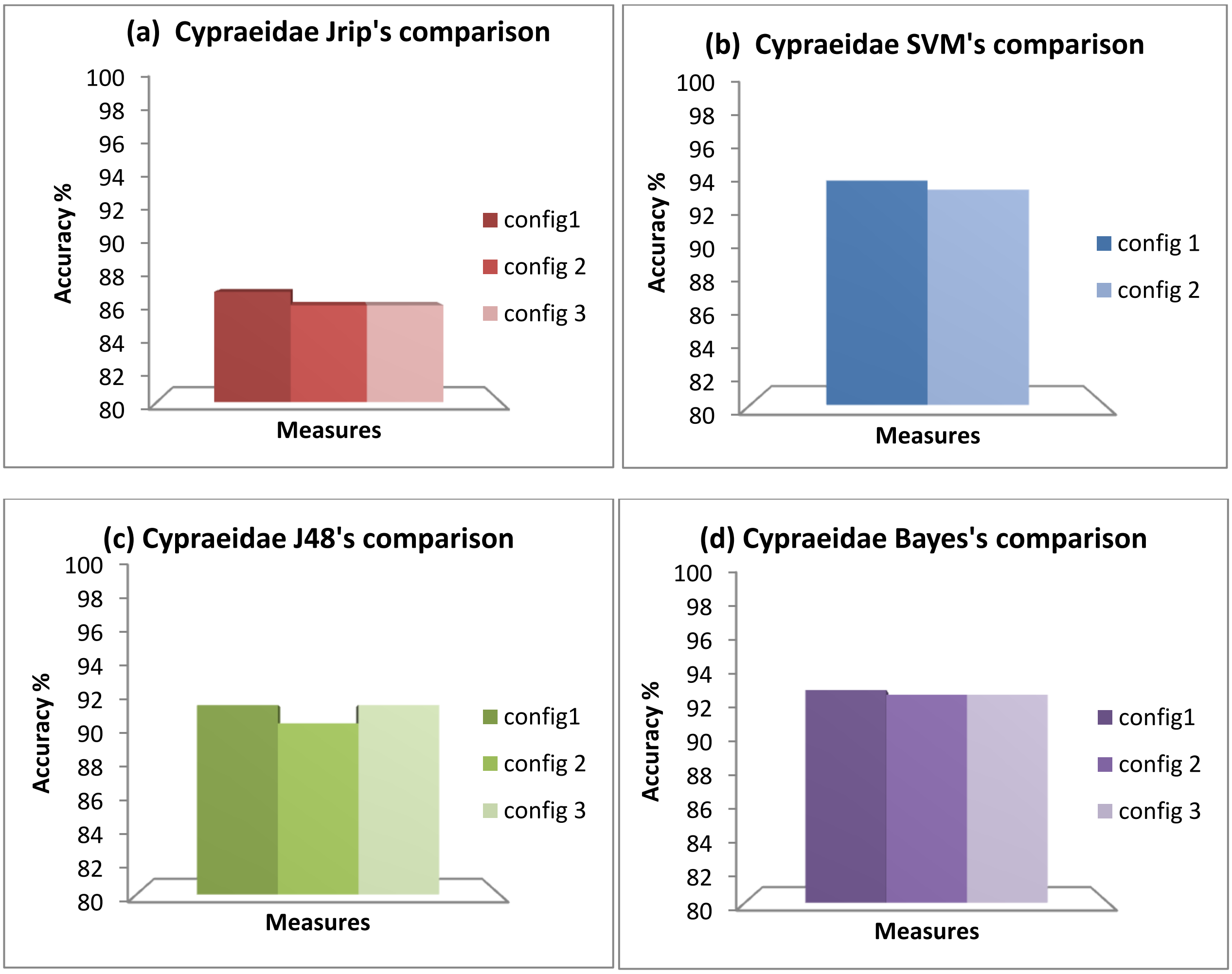

Figure S12 Results of comparative analysis performed on Cypraeidae empirical data set: (a) Configurations comparison of rules-based method Jrip; (b) Configurations comparison of function-based method SVM; (c) Configurations comparison of tree-based method J48; (d) Configurations comparison of Bayesian-based method Naïve Bayes.

Drosophila

Table S3 Drosophila parameters configuration (see Table 3 for explanation of the default parameters configuration)

#Configuration	Jrip	SVM	J48	Naïve Bayes
**1**	default	default	default	default
**2**	pruning=FALSE	Standardization	reduce error pruning=TRUE	kernel estimator=TRUE
**3**	optimization=10	kernel= RBF kernel	use Laplace=TRUE	supervised discretization=TRUE
**4**	folds=20	built logisticmodel	sub tree raising=FALSE	-
**5**	optimization=5 folds=5	kernel=normalized polyKernel	unpruned=TRUE	-

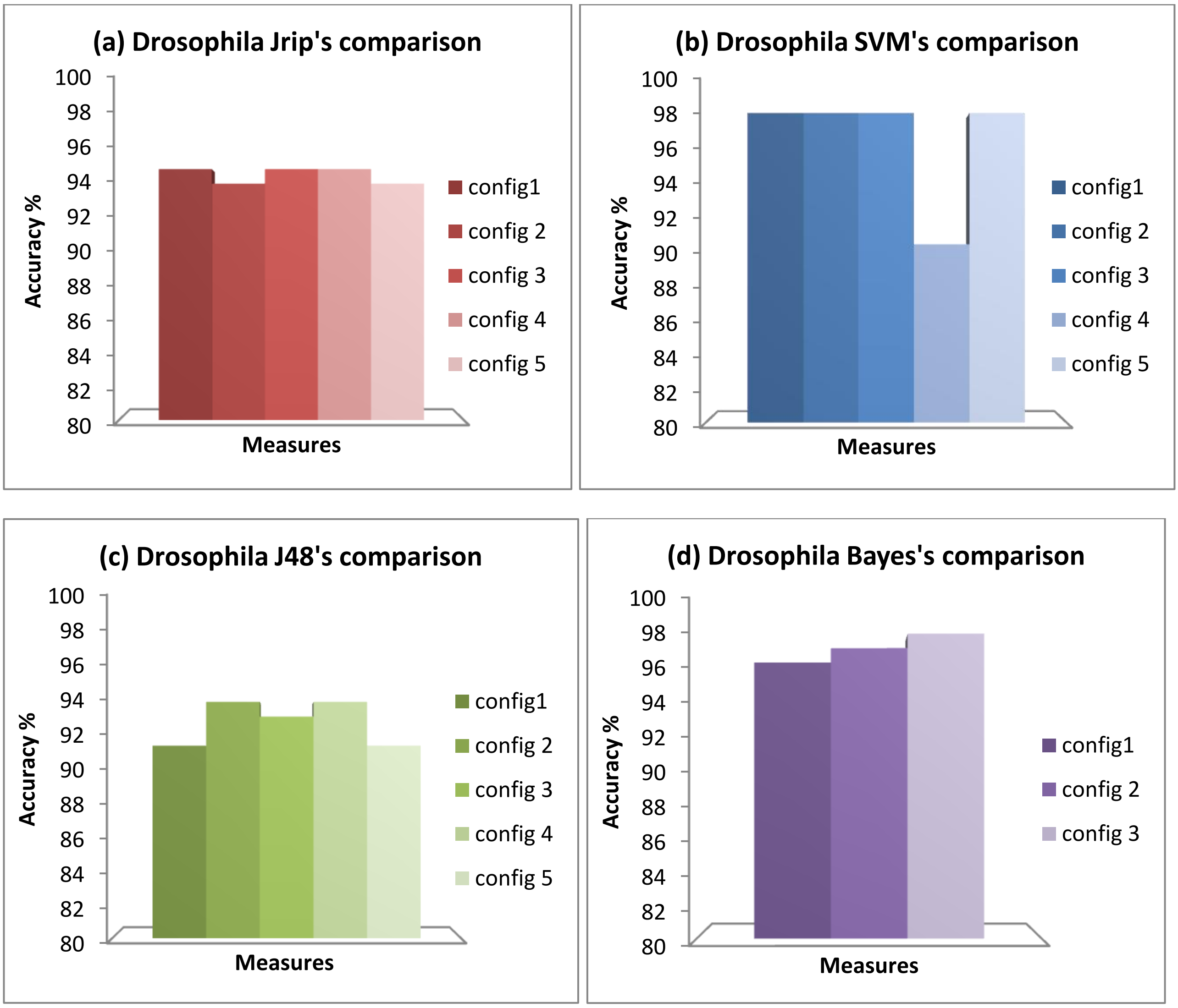

Figure S13 Results of comparative analysis performed on Drosophila empirical data set: (a) Configurations comparison of rules-based method Jrip; (b) Configurations comparison of function-based method SVM; (c) Configurations comparison of tree-based method J48; (d) Configurations comparison of Bayesian-based method Naïve Bayes.

Inga

Table S4 Inga parameters configuration (see Table 3 for explanation of the default parameters configuration)

#Configuration	Jrip	SVM	J48	Naïve Bayes
**1**	default	default	default	default
**2**	pruning=FALSE	filter Type=standardize training data	reduce error pruning=TRUE	kernel estimator=TRUE
**3**	optimization=5	kernel=RBF kernel	seed ≠ 1	supervised discretization=TRUE
**4**	-	built logistic model	-	-

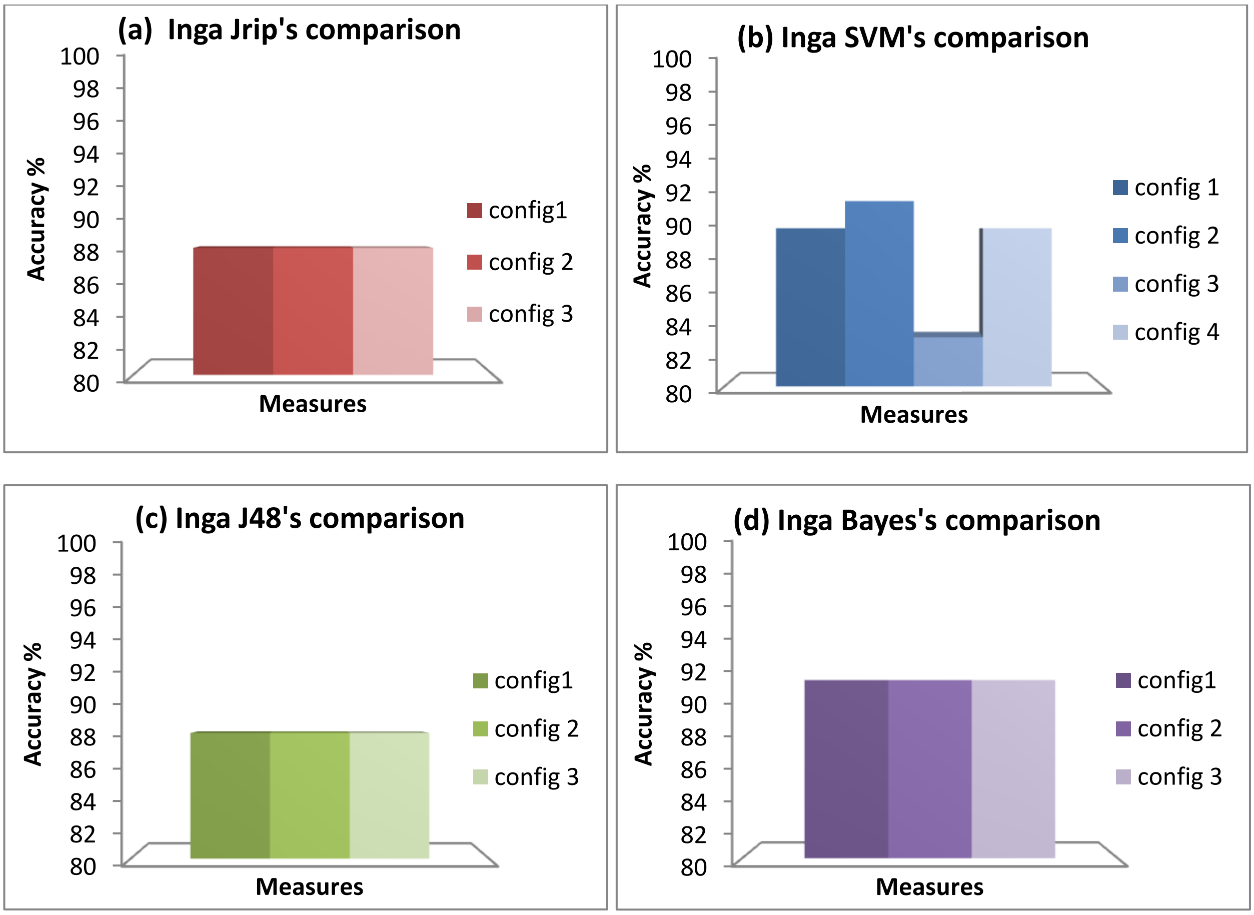

Figure S14 Results of comparative analysis performed on Inga empirical data set: (a) Configurations comparison of rules-based method Jrip; (b) Configurations comparison of function-based method SVM; (c) Configurations comparison of tree-based method J48; (d) Configurations comparison of Bayesian-based method Naïve Bayes.
