# Supplementary material for: Supervised DNA Barcodes species classification: analysis, comparisons and results
Source: BioData Min. 2014 Apr 11;7:4. doi: 10.1186/1756-0381-7-4 (PMC4022351; doi:10.1186/1756-0381-7-4)
Supplement: Additional file 2 — Additional results and tables of the classification analysis on empirical and synthetic datasets. [file 1756-0381-7-4-S2.pdf]

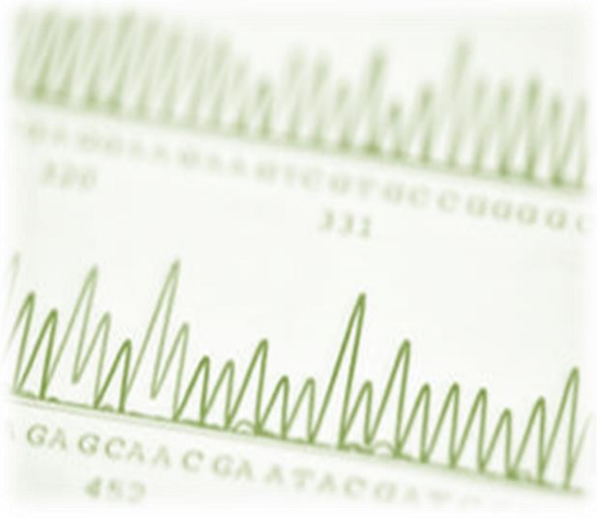

Supervised DNA barcodes species classification: analysis, comparisons and results

Tutorial

Emanuel Weitschek, Giulia Fiscon, and Giovanni Felici

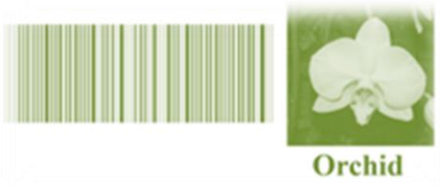

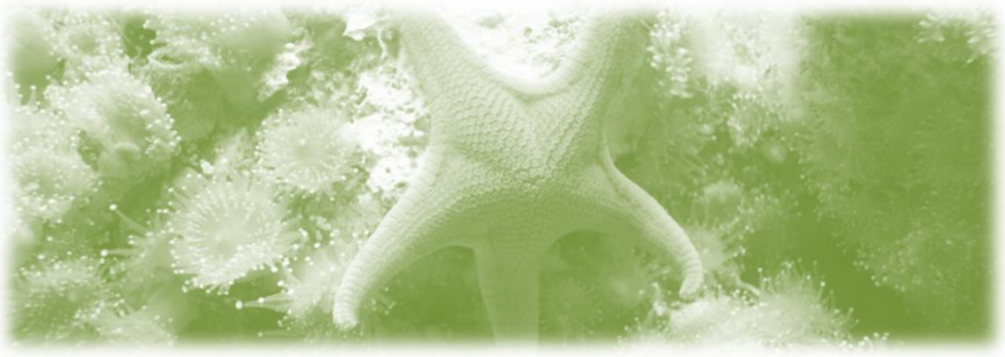

Citations

If you use this procedure please cite:
Weitschek E, Fiscon G, and Felici G
Supervised DNA Barcodes species classification: analysis, comparisons and results
BMC BioData Mining

Contents

Introduction 2
  About the tutorial 2
  Supervised Machine Learning 2
Supervised Machine Learning procedure steps 2
DNA Barcode sequences download 3
Sequences alignment 3
Reference and query split 3
Installation 3
  JAVA 3
  Weka 3
  Fasta to Weka Converter 4
Executing the programs 4
  1. Fasta2Weka Converter 4
    Start screen 4
    Input 4
    Run Fasta to Weka Converter 4
  Weka 5
    Weka Explorer Start screen 5
    Input 6
    Classifiers 7
Data examples 9
Contacts 9

Introduction

About the tutorial

This manual is intended for all users who want to learn how to use the Weka Machine Learning software and the special FASTA sequences converter for DNA Barcodes classification. It is process-based and explains how to achieve a specific objective on a step-by-step basis.

Supervised Machine Learning

The goal is to assign an unknown specimen to a known species starting from its DNA Barcode sequence.
The supervised machine learning classification problem may be formulated in the following way. Given a reference library composed of DNA Barcode specimen sequences of known species and a collection of unknown DNA Barcode sequences (query set) recognize the latter into the species that are present in the library
To obtain reliable results:

- the query set has to contain only specimens from the same species that are present in the reference library;
- the reference set has to contain a sufficient number of specimens sequences for each species (our experiments show that at least 4 specimens per species are necessary to obtain a reliable classification rate);
- the sequences of each species in the reference set have to include possibly all the nucleotide polymorphisms (variations).

The user has to provide as input a training set (reference library) containing specimens with a priori known species membership.
Based on this training set, the software computes the classification model.
Subsequently, the classification model can be applied to a test set (query set) which contains specimens that require classification.
The test set can contain query specimens with unknown species membership or, alternatively, specimens that also have a priori known species membership, allowing verification of the specimen classifications.

Supervised Machine Learning procedure steps

The following steps are necessary to perform a DNA Barcodes sequences classification with the suggested supervised machine learning procedure. These steps are going to be thoroughly explained in the next sections of this tutorial.

1. Sequences acquisition;
2. Sequences alignment;
3. Reference and query split;
4. Software download and installation;
5. Sequences conversion;
6. Classification

DNA Barcode sequences download

To run the experiments, firstly you have to acquire the DNA Barcode FASTA sequences, for example you can download them from dmb.iasi.cnr.it/supbarcodes.php and BOLD system (www.boldsystems.org/).

Sequences alignment

If not already performed by the data download system, please align the acquired sequences obtaining DNA Barcode sequence alignments in the standard FASTA format (query and reference).

Reference and query split

The sequences have to be split in reference and query sets. See the supervised machine learning section above for further explanation.

Installation

JAVA

Weka and the special FASTA converter strictly require a working JAVA Virtual Machine (VM) installed. Before going on download and install the free Oracle JAVA Runtime Environment from www.java.com/getjava/.

Weka

You can download and install Weka from www.cs.waikato.ac.nz/ml/weka/downloading.html.
Several versions for the most common operating systems are available, please chose Windows x86 if you have Windows 32 bit, Windows x64 for Windows 64 bit, MacOsX, or Linux. Please chose the versions without Java VM according to your operating system.
Then follow the install wizard by agreeing to the license agreement and by clicking "next" to the following steps.

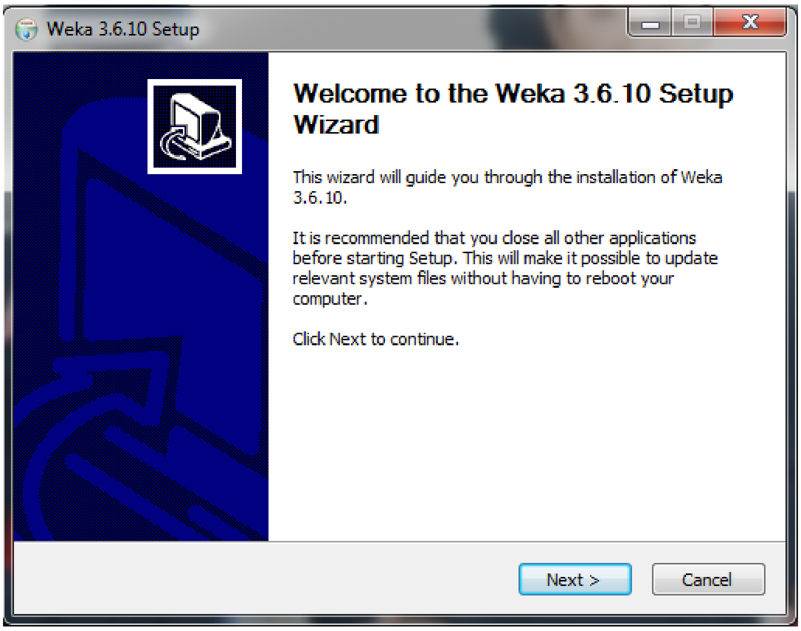

Weka installation wizard

Fasta to Weka Converter

You can download and unzip an integrated multi-platform (Windows, Linux and MacOS) Java software FASTA to Weka converter from dmb.iasi.cnr.it/supbarcodes.php ("fasta2weka.zip"), that transforms the DNA Barcode FASTA sequences to the suitable ARFF Weka format.

Executing the programs

1. Fasta2Weka Converter

Go to the directory where you extracted the Fasta2Weka archive and execute fasta2weka.bat for Windows and fasta2weka.sh for Linux/MacOs.

Start screen

Once you executed the converter, the main screen of the converter will appear.

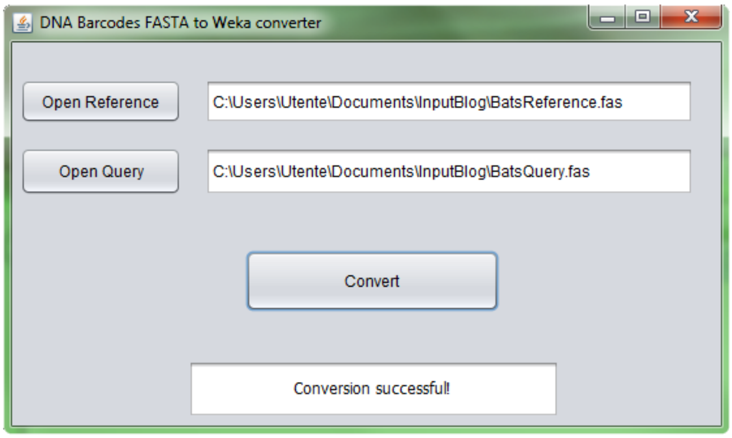

Converter start screen

Input

The input of Fasta2Weka converter is the standard FASTA format of the DNA Barcode Sequences. The heading line of each sequence is composed by the starting character ">" and the "specimen id" and "species name field" separated by the pipe character "|" (eg: ">E3434243 | squalus edmundsi").

Run Fasta to Weka Converter

To succeed the conversion from FASTA to ARFF format you have to:

- click the **Open Reference** button and select the reference sequence (FASTA) to be converted;
- click the **Open Query** button and select the query sequence (FASTA) to be converted (optional if you want to convert only the reference set) ;
- click the button **Convert** to start the conversion of both reference (and query) sequences.

If the conversion succeeds , the message "Conversion Successful" will be displayed.
The output files will be saved in the same directory of the input files.

Weka

Go to the start menu and execute the Weka GUI. The following screen will appear. Please choose the "Explorer" Application.

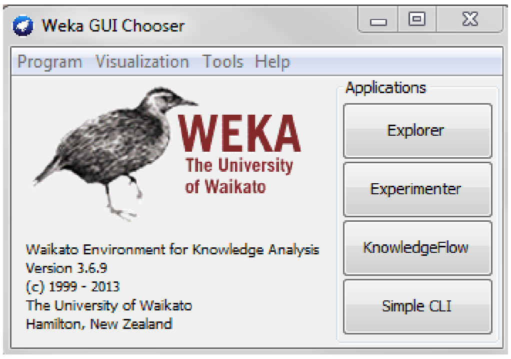

Weka GUI start window

Weka Explorer Start screen

The main screen in Weka Explorer is the starting point for all actions. The **Weka Explorer start screen** has different sections: Preprocess, Classify, Cluster, Associate, Select Attributes, and Visualize. Please go to the "Preprocess" section in order to load the datasets to be analyzed.

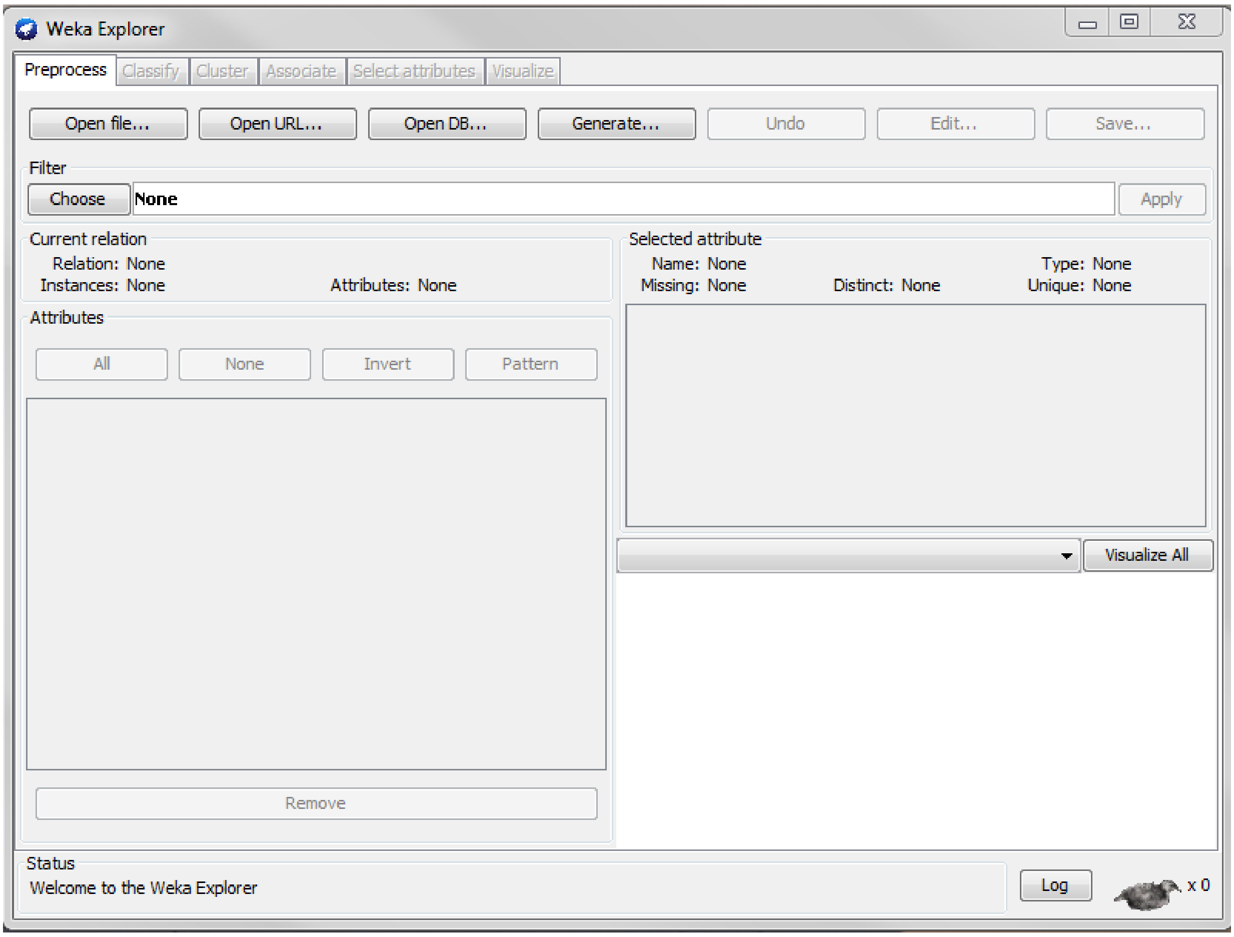

Weka Explorer start screen

Input

The input format of Weka are the DNA Barcode sequences in ARFF format. We remind that the common FASTA DNA Barcodes sequences need to be converted. The "Open File" button allows user to select the ARFF datasets to be opened.

- Click the **Open File** button in the Weka Explorer start screen and the **file selection window** appears.

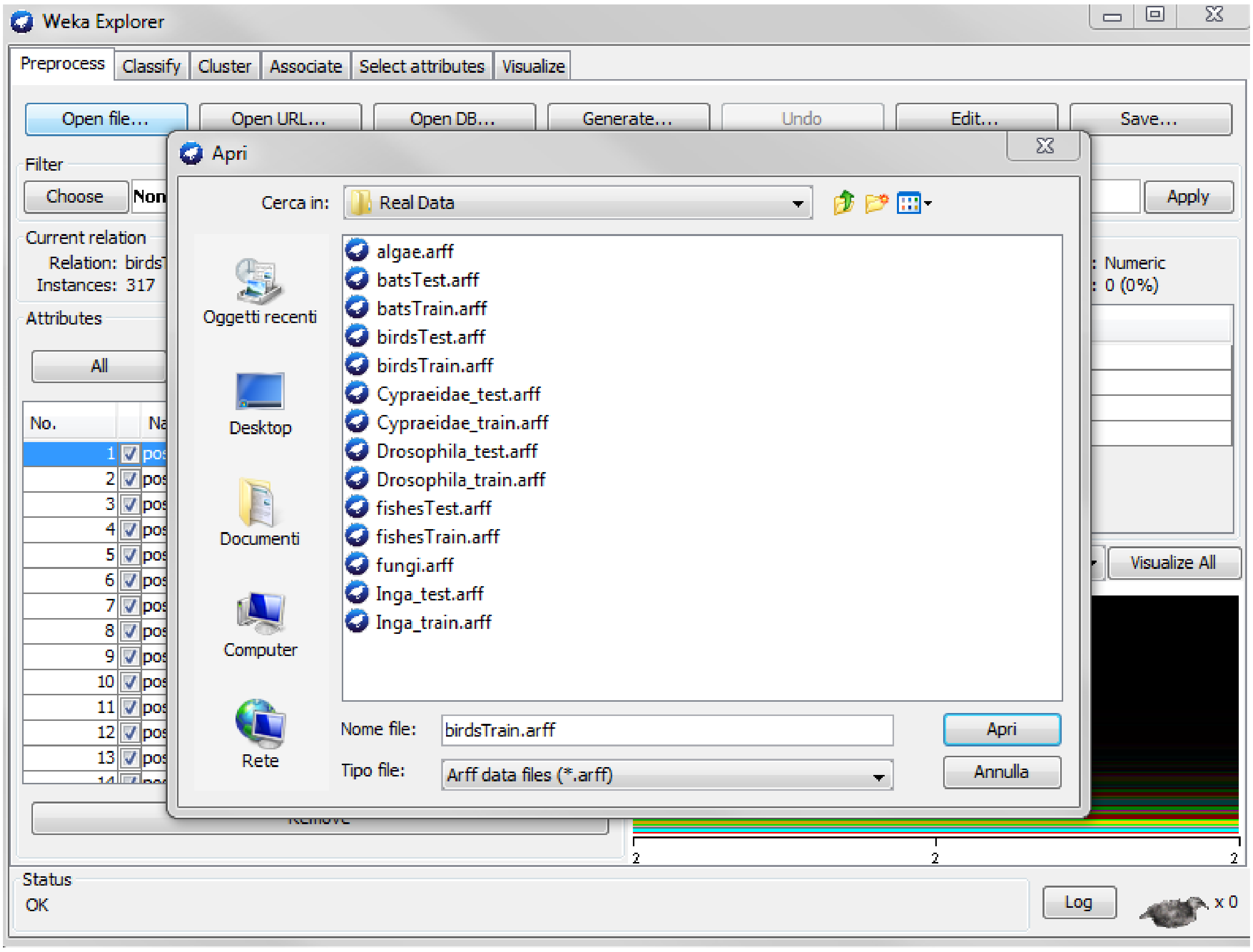

File selection window

- Select the .arff file of reference set (training set) that you want to open and click the Open button: the arff file of the reference set is now open and the number of instances and attributes is displayed.

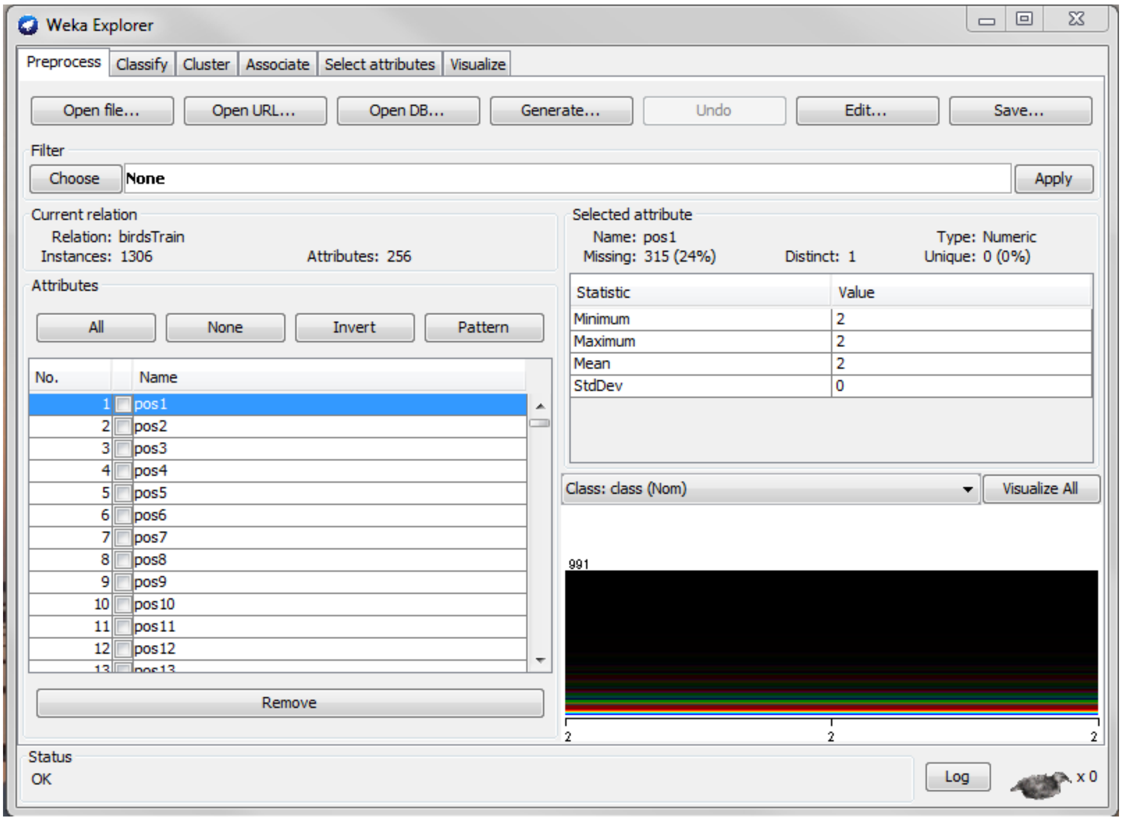

Input selected file window

Classifiers

Once you open a reference file, the section “Classify” is activated and the “choose” button allows to select the classifier that you want to use.

- Select the classifier family and type.

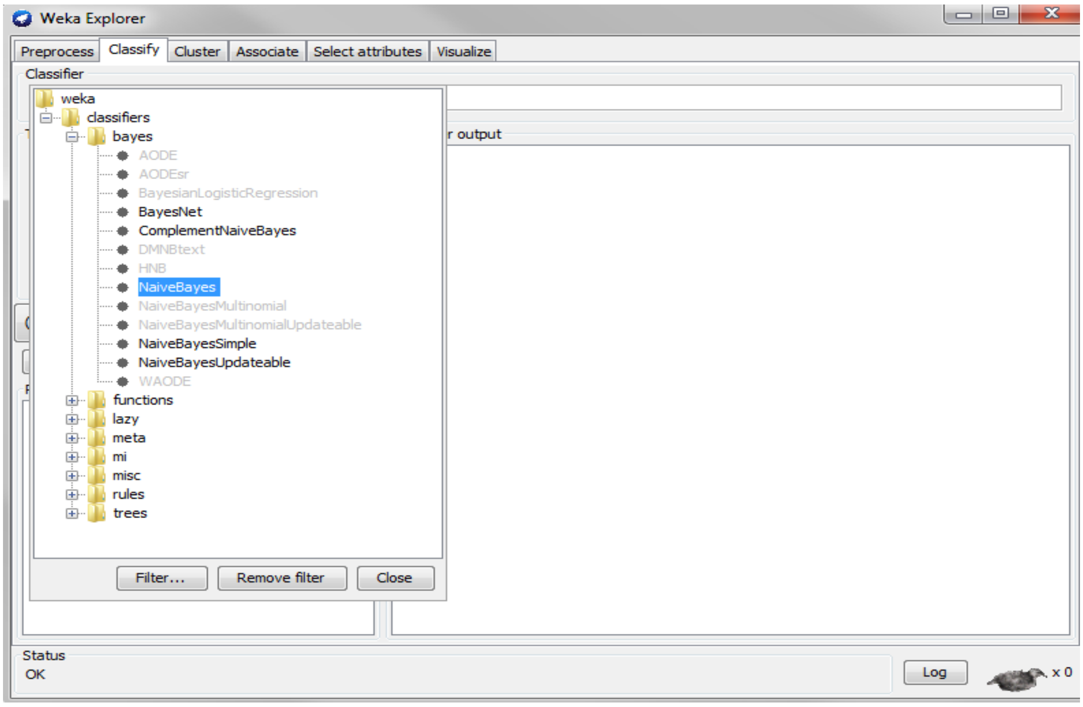

Classifier selection window

- Optionally, click to **Supplied test set** in order to select the dataset to be used as query set. Alternatively, you could use only one file, indicating the Percentage split or cross-validation.

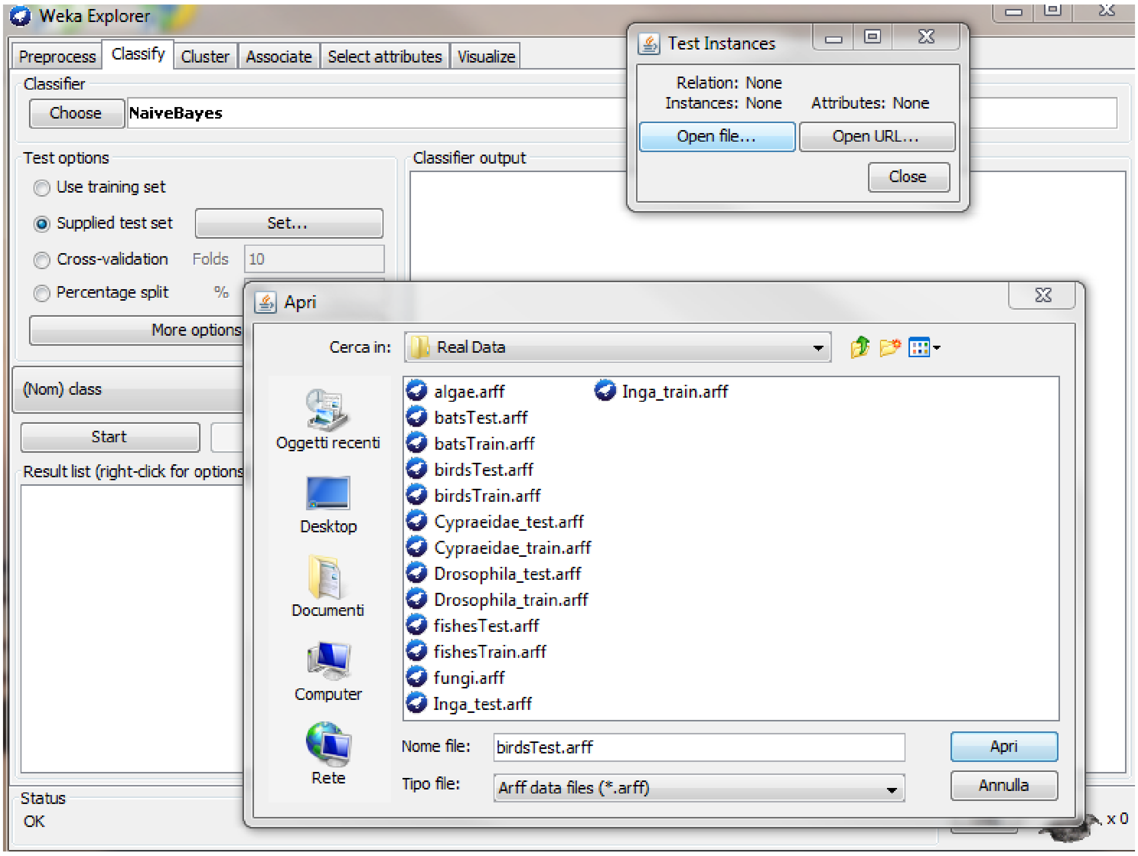

Test set selection window

- If you have unknown barcodes sequences in the query set and you want the specimen to species assignments, through the **More options** button select the "Output prediction" option.

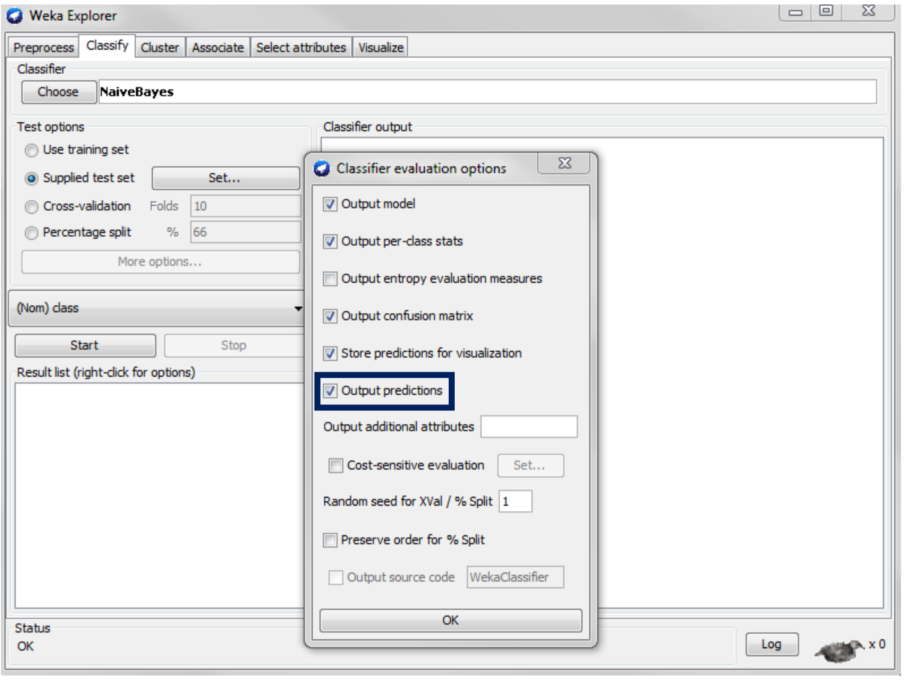

Highlighting output prediction option

- Click the **Start** button to run the experiment.

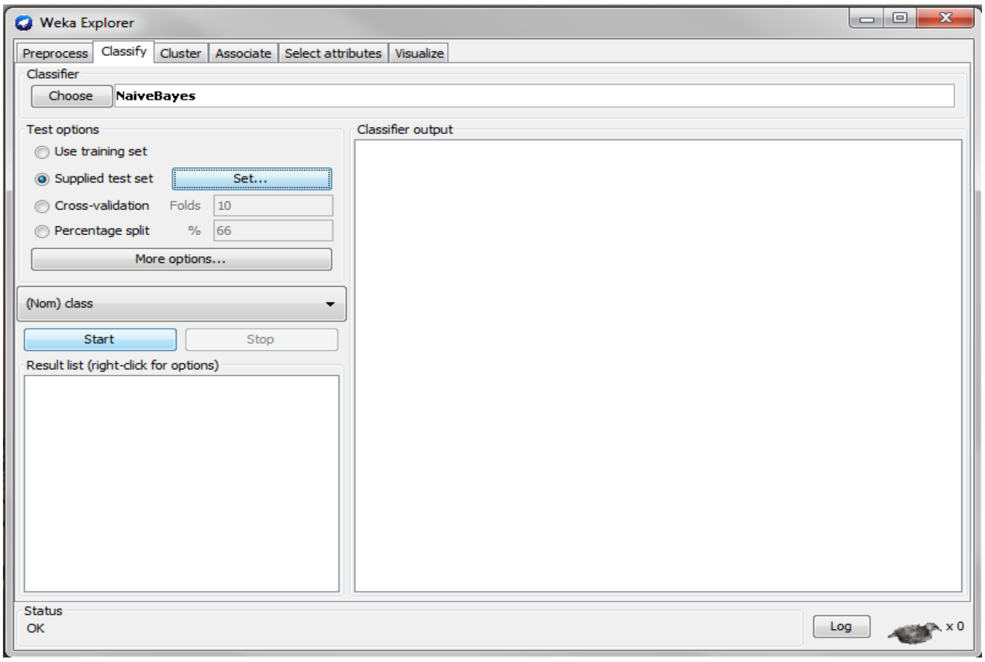

Start classification window

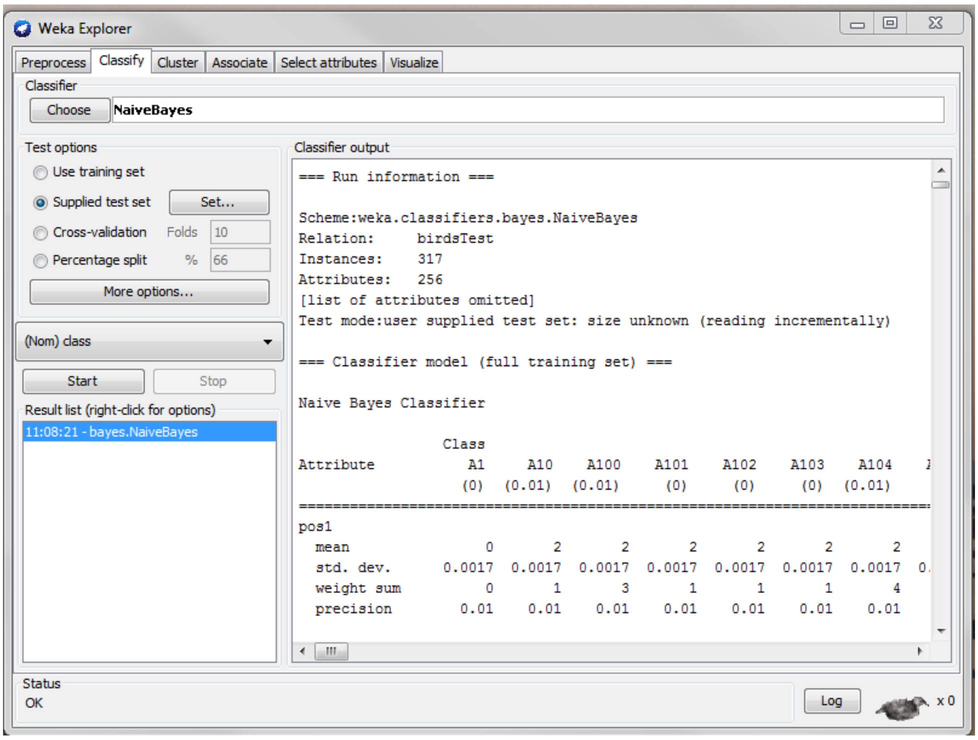

Classifier output window

Data examples

The website http://dmb.iasi.cnr.it/supbarcodes.php contains the Fasta2Weka converter software package and some example DNA Barcode sequences data sets.

Contacts

Please contact Emanuel Weitschek (emanuel.weitschek@iasi.cnr.it) for comments and questions.
